# The Home-Cage Automated Skilled Reaching Apparatus (HASRA): Individualized Training of Group-Housed Mice in a Single Pellet Reaching Task

**DOI:** 10.1523/ENEURO.0242-20.2020

**Published:** 2020-10-12

**Authors:** Gilles Salameh, Matthew S. Jeffers, Junzheng Wu, Julian Pitney, Gergely Silasi

**Affiliations:** Department of Cellular and Molecular Medicine, Faculty of Medicine, University of Ottawa, Ottawa, Ontario K1H 8M5, Canada

**Keywords:** behavior, forelimb, motor, mouse, reaching, recovery

## Abstract

The single pellet reaching task is commonly used in rodents to assess the acquisition of fine motor skill and recovery of function following nervous system injury. Although this task is useful for gauging skilled forelimb use in rodents, the process of training animals is labor intensive and variable across studies and labs. To address these limitations, we developed a single pellet reaching paradigm for training and testing group housed mice within their home cage. Mice enter a training compartment attached to the outside of the cage and retrieve millet seeds presented on a motorized pedestal that can be individually positioned to present seeds to either forelimb. To identify optimal training parameters, we compared task participation and success rates between groups of animals that were presented seeds at two different heights (floor vs mouth height) and at different intervals (fixed-time vs trial-based). The mouth height/fixed interval presentation style was most effective at promoting reaching behavior as all mice reached for seeds within 5 d. Using this paradigm, we assessed stroke-induced deficits in home-cage reaching. Following three weeks of baseline training, reaching success rate was ∼40%, with most trials performed during the dark cycle. A forelimb motor cortex stroke significantly decreased interaction with presented seeds within the first 2 d and impaired reaching success rates for the first 7 d. Our data demonstrate that group-housed mice can be efficiently trained on a single pellet reaching task in the home cage and that this assay is sensitive to stroke induced motor impairments.

## Significance Statement

We developed an automated apparatus and single pellet reach training paradigm for group housed mice within the home cage. This task allows individualized training progression and handedness of presentation for each mouse, minimizes stress induced by experimenter interaction, and enables experiments and data collection on a massive scale, previously impossible because of the demands of one-on-one behavioral testing. We demonstrate an optimized set of task parameters and the utility of this apparatus for assessing lesion-induced motor impairments. Herein, we provide an open-source, scalable, platform for training and assessing skilled reaching in mice with minimal need for experimenter handling that is equivalent to gold-standard, manual single pellet training paradigms.

## Introduction

The single pellet reaching task is the gold standard for assessing forelimb motor function in rodents. The task requires animals to reach through a slot at the front of a training chamber and retrieve a food pellet that is presented either on a shelf or pedestal (Whishaw and Pellis, 1990; Whishaw, 2000; Farr and Whishaw, 2002; Whishaw et al., 2017). Quantifying this behavior provides several metrics of skilled forelimb use that are commonly used to assess motor learning and gauge impairments after neuronal injury. For example, the rate of task acquisition can be used to assess how various genetic manipulations impact skilled motor learning (Gu et al., 2017; Ueno et al., 2018), while endpoint measures such as reaching success rate are sensitive to acute changes in brain function induced by injury or temporary inactivation (Silasi et al., 2008; Brown and Teskey, 2014; Guo et al., 2015; Clark et al., 2019). Quantification of movement kinematics can also be performed to assess subtle changes in reaching behavior, such as the emergence of compensatory movements after injury (Gharbawie et al., 2005a,b) or alterations in movement subcomponents induced by pharmacological manipulations (Metz et al., 2003).

Although the single pellet reaching paradigm is widely used, there are two main challenges that limit the utility of the task. First, the majority of training requires the experimenter to work with the animals one at a time, limiting the amount of individual training time and number of animals that can be included in experiments. Second, individual differences between experimenters may affect the quality of training and overall reaching success rate that is achieved (Fouad et al., 2013). Because of these challenges, there has been great interest in automating the training process with hopes of increasing throughput and minimizing variability. Several studies have shown that automated training is in fact feasible and has measurable benefits (Fenrich et al., 2015; Ellens et al., 2016; Gu et al., 2017; Torres-Espín et al., 2018; Hubbard et al., 2019). For example, presenting pellets using a motorized delivery arm reduced variability in success rate between and within experiments compared with manually presented pellets (Torres-Espín et al., 2018). Allowing animals to self-initiate trials by activating a sensor within the testing chamber (Wong et al., 2015; Ellens et al., 2016) can be used to further reduce the need for interaction with the experimenter, thus increasing the number of subjects that can be trained simultaneously by running multiple training chambers in parallel. Although this form of automated training results in reaching success rates that are similar to manual training, one drawback is that animals must still be removed from the home cage by the experimenter, transferred to a testing apparatus, and tested during a specific time of day (typically during the light cycle). In addition to being labor intensive, handling the animals induces a persistent stress response even when habituated (Balcombe et al., 2004), interrupts their rest cycle, and produces periods of social isolation. An alternate approach is to perform training and testing within the home cage (Woodard et al., 2017; Silasi et al., 2018; Bollu et al., 2019; Erskine et al., 2019). In addition to increasing the number of animals that can be tested simultaneously in a single apparatus, home cage testing facilitates self-initiated task participation during the natural active (dark) cycle of the animals without having to reverse the light cycle of the animal facility.

Based on these advantages, we developed a home-cage automated skilled reaching apparatus (HASRA) that can be used to train group-housed mice to a reaching success rate similar to manual training using the single-seed variant of the task (Xu et al., 2009). This minimizes stress to the animals by limiting the presence of experimenters during testing. We systematically modulated two key training parameters, pellet presentation height and timing of trial presentation, to determine optimal training parameters. Using this system, we then quantified reaching impairments in a group of mice that received a photothrombotic motor cortex stroke.

## Materials and Methods

### HASRA

Rodents display strong lateralization of limb use, preferring to reach with either their left or right hand (Whishaw, 1992). Furthermore, training in the single pellet task typically involves initially allowing retrieval of pellets in a proximal position with either the tongue or hand and progressively shaping the rodent to begin using only the hand to retrieve pellets from a more distal position (Whishaw et al., 2008). In order to accommodate these strategies among group-housed mice, it was crucial that our device be able to deliver the pellet in positions that were individualized to each animal’s hand preference and training stage.

**Figure 1.**
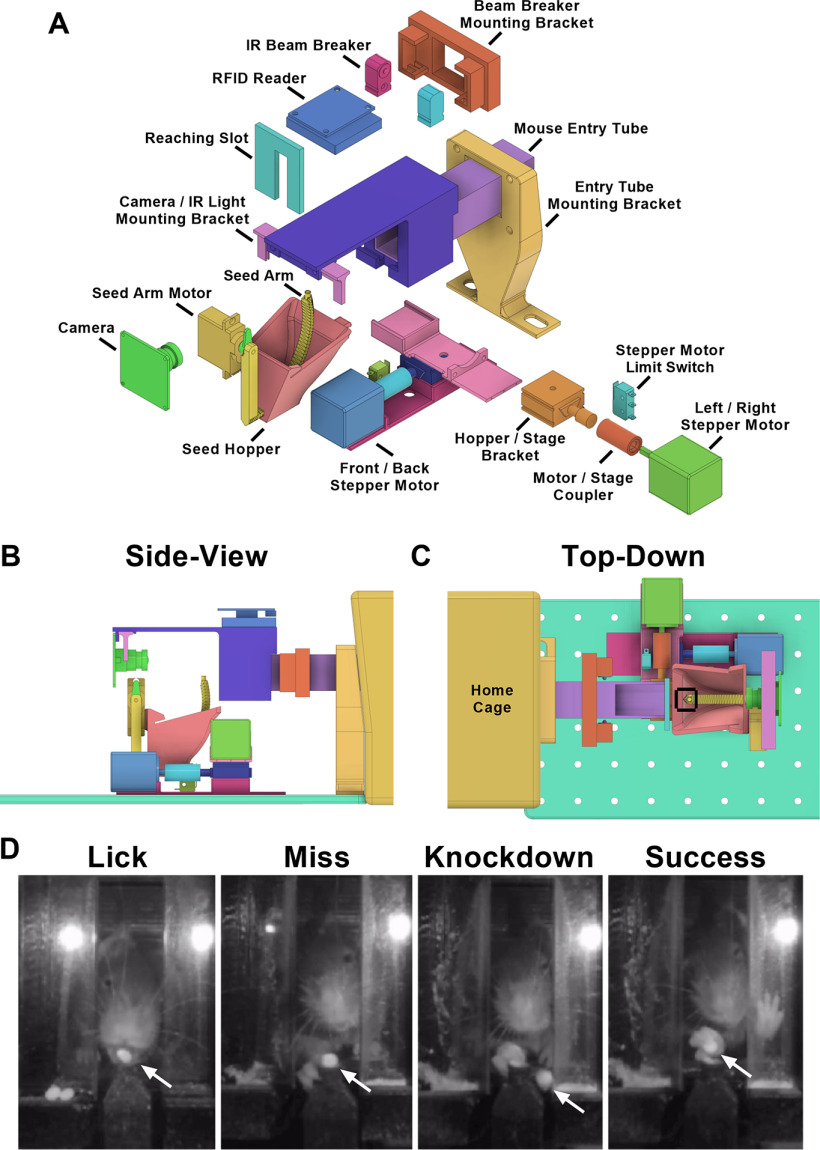
HASRA. ***A***, Exploded view of apparatus, showing each part of the assembly. ***B***, Side view of apparatus, showing relative positions of reaching compartment, seed arm, camera, and RFID tag reader for identifying mice. ***C***, Top-down view of front wall, stage motors and range of motion in which a seed could be presented (within black bounding box). ***D***, Representative images taken from wall-facing camera showing a mouse performing each type of event that was classified. Miss, knock-down, and success represented types of attempts to retrieve the pellet using the hand. This perspective was used for scoring data on mouse reaching performance. Location of seed indicated with white arrow in each image.

Two stepper-motors (Pololu, 1205) controlled the proximal-distal and medial-lateral positioning of a seed presentation arm and hopper apparatus inspired from a previously published design (Torres-Espín et al., 2018). The presentation arm’s movement in both axes could be adjusted within a ∼1-cm range relative to the front slot (3.5 cm tall × 1.0 cm wide; Fig. 1*A*) that the mice reached through. A servo motor (Hitec, HS-82MG) mounted to the seed hopper controlled the speed and height of the seed presentation arm relative to the entry tube’s floor level. Upon activation, the seed delivery arm was initially immersed in the seed hopper and traveled up to its set height. A ∼1-mm-deep well in the tip of this arm ensured presentation of a single seed. All motors were controlled by an Arduino Nano (Arduino, 7630049200173). Most parts, excluding electronics, were 3D printed in the laboratory using a fused deposition modeling printer (FlashForge 3D printer, Creator Pro). The front wall reaching slot was cut from acrylic using an Epilog laser cutter. Up-to-date part listings and instructions to build the device (including Python/Arduino code, 3D printing STL files, and wiring diagrams) can be found at https://github.com/SilasiLab/HASRA. An archived version of these files at the time of publishing can be found attached to this article (Extended Data 1).

10.1523/ENEURO.0242-20.2020.ed1 Extended Data 1 Archive of assembly instructions, Python/Arduino code, 3D printing STL files, and wiring diagrams. See “homecage assembly manual.pdf” and “README.md” contained in archive for more details. Download Extended Data 1, ZIP file.

### Radio frequency identification (RFID) tag insertion and HASRA activation

All mice were subcutaneously implanted with a unique RFID tag (Sparkfun, SEN-09416) enabling individual identification. Animals were briefly (1–2 min) anesthetized using 4% isoflurane in room air and injected between the scapula with a sterile RFID tag. Upon regaining mobility, mice were introduced to the new home cage and immediately had access to the HASRA.

A 10-cm acrylic tube (2.54 × 2.54 cm) sized to fit one mouse at a time was attached to the home cage, leading to a front wall with a slot through which animals could reach for millet seeds (Chen et al., 2014). The entry of a mouse into the tube initiated a trial by interrupting an infrared beam projecting across the width of the tube (Adafruit, 2167). Our custom Python script sampled the RFID reader (Sparkfun, SEN-11827) mounted on top of the tube (Fig. 1*B*) to identify the animal and triggered the motorized hopper and seed presentation arm to move to a user defined position and present a millet seed (Fig. 1*C*). Each presentation was recorded using a video camera directly facing the reaching slot. When the mouse exited the tube (detected by closed IR breaker), the seed hopper and presentation arm returned to a home position and video recording was discontinued.

### Video recording and seed detection

Detection of the mouse by the RFID tag reader activated a video camera (ELP, USBFHD08S-LC1100) equipped with an adjustable focus lens, which recorded at 60 fps until the mouse exited the tube. Two infrared light sources (HonYan, 191094177157) flanked the camera lens to enable recording during the dark cycle. A Python script automatically uploaded videos (where a minimum of one seed was presented) to a Google Drive folder corresponding to the mouse’s RFID tag (Movie 1). This enabled the experimenter to remotely monitor mouse activity and quantify videos without overwhelming local storage capacity. Videos where a mouse exited the tube before at least one seed could be presented were automatically deleted.

Movie 1. Example video showing a mouse engaging with the seed delivery arm to retrieve millet seeds. 10.1523/ENEURO.0242-20.2020.video.1

A neural network was used to continually scan the video feed for the presence/absence of a seed. This neural network was a variation of the MobileNetV2 architecture (Sandler et al., 2018). To train the network, a dataset of *N* = 5482 video frames was collected from HASRA. These frames were manually labeled as either containing a seed or not containing a seed. In an effort to increase the variability of this small training dataset (and therefore increase the ability of the trained network to generalize to novel examples), these labeled frames were subjected to standard supervised machine learning dataset augmentation techniques including rotations, scaling and brightness adjustments. The dataset was then partitioned into validation and training partitions containing 10% and 90% of the total frames, respectively. The network was then trained on the training partition for 50 epochs with a batch size of 32. The trained network reached an accuracy of 99.08% on the validation partition. The network was then deployed to the HASRA where it was used to detect the presence/absence of seeds in real time. This real-time detection was used to trigger the presentation of a new seed whenever no seed was detected for ≥4 s.

Automated detection of successful seed presentation also enabled a method to benchmark the mechanical success rate of presentation for a given arm (successful presentation rate was checked daily, and maintenance was performed when successful presentation dropped below 90%). Maintenance activities consisted of filling the hopper with seeds, removing crumbs from the ∼1-mm-deep well at the tip of the motorized arm and bottom of the hopper using a brush and completely replacing the arm with a new one if presentation rate could not be otherwise improved. Additionally, if mice had caused any damage to the front wall of the reaching slot by chewing then this would be replaced with a new front wall. These maintenance activities required the HASRA to be taken offline for no more than a few minutes. No other mechanical issues were observed during these experiments, although we have observed that it is possible for the motors and mechanical stages to eventually fail after months of continual use, at which point they can be easily replaced.

### Validation experiment design

A total of 27 Thy1-ChR2-YFP mice (The Jackson Laboratory, stock #007612), aged between 44 and 80 d old, were housed in groups of three to five on a 12/12 h light/dark cycle. Standard mouse chow was restricted to 1 g/mouse/d, meaning that for a cage of five mice, we provided 5 g of chow per day. Mice were able to supplement their diet with millet seeds *ad libitum* by successfully using the HASRA. Although we were not able to track the exact amount of chow that each mouse consumed, we broke the pellets into small pieces and spread them throughout each cage to allow all mice a chance to forage and obtain it. Water was accessible *ad libitum*. Animals were weighed weekly to monitor their body weight. All procedures were performed in accordance with our Animal Care Committee and complied with Canadian Council on Animal Care (CCAC) guidelines.

In experiment 1, we determined the optimal seed arm height (floor height vs mouth height) and presentation style (continuous 5-s cycling vs stationary presentation until seed no longer detected) to maximize task engagement, progression from licking to reaching behavior, and reaching success rate within the HASRA. This was conducted over a two-week period with four groups: (1) floor height with continuous cycle (low cycle, *n* = 5 female mice); (2) floor height with stationary presentation (low single, *n* = 5 female mice); (3) mouth height (0.8 cm above the floor) with continuous cycle (high cycle, *n* = 5 female mice); and (4) mouth height with stationary presentation (high single, *n* = 5 female mice).

In experiment 2, a second group of mice using the factorial combination with the greatest success in experiment 1 (high cycle) was trained over a three-week period (*n* = 7 male mice). This replicated and extended the training curve obtained in experiment 1. At the conclusion of training, mice were randomly assigned to receive either a photothrombotic stroke (*n* = 4) or a sham surgery (*n* = 3). Mice were allowed continual access to the HASRA for 7 d following stroke, with their performance quantified to assess the sensitivity of this task to stroke-induced impairments of skilled reaching and grasping.

### Daily qualitative scoring

Daily qualitative scoring was used to describe the animals’ performance and engagement with the HASRA. Approximately five videos per mouse were evaluated each day and attributed a score from 1 to 4. A score of 1 was credited to a mouse not showing any licking or reaching behavior. If an animal was seen only licking the seed displayed in front of the wall at least once, it received a score of 2. A score of three represented a combination of licking and reaching behavior. Finally, a mouse that solely reached for the seed obtained a score of 4. This score was used to assess the animals’ behavior and inform the experimenter on each animal’s training stage for the adjustment of the arm’s position each day.

### Training stages

#### Stage 1

Upon initial introduction to the HASRA, the presentation arm was set to deliver seeds at the closest distance possible (0.4 cm from front wall) and in the center of the front wall slot to encourage licking of the seed. This was intended to habituate animals to the HASRA, and millet seeds presented outside the front wall. An animal moved to stage 2 only if it was observed using its tongue or hand to successfully retrieve a seed.

#### Stage 2

Once seed retrieval was observed, the presentation arm was progressively moved further away from the front wall (∼0.4 cm/d up to a maximum of 1.5 cm) until the animal could no longer retrieve the seed by licking. This stage was intended to eliminate licking behavior and train animals to use their hand to reach and grasp the seed. An animal completed this stage when it frequently used its hand for seed retrieval.

#### Stage 3

Once an animal retrieved the seed by reaching and grasping with its hand, seeds were presented with a left or right offset relative to the edge of the slot opposing the preferred hand (∼0.5 cm off-center) as is done during manual training (Farr and Whishaw, 2002). During this final stage animals developed success at skilled reaching by repetitively performing the task using their preferred hand.

### Reaching success rate

The reaching success of each animal was quantified by scoring 20 events per mouse at each desired time point (weekly in experiment 1, every second day in experiment 2). Each reaching event was classified into one of four categories: lick, miss, knock-down, success (Fig. 1*D*). A “lick” was any pellet retrieval that was completed using the tongue and not the hand. An “attempt” was defined as any action where the mouse moved its hand beyond the front wall slot. Several subcategories of attempt were also defined (miss, knock-down, success). A “miss” was defined as an attempt where the movement of the hand did not disrupt the position of the seed. A “knock-down” was defined as an attempt that displaced the seed from the arm but did not successfully retrieve it. A “success” was an attempt in which the seed was grasped and retrieved using the hand to the animal’s mouth. Success rate was calculated as: (number of success/number of attempts) × 100.

### Photothrombotic stroke induction

Mice were anaesthetized using 4–5% isoflurane and secured in a stereotaxic frame using ear bars. An incision was made along the midline of the scalp to expose the skull. A total of 100 mg/kg of rose bengal (Sigma-Aldrich, 330000) diluted in PBS was injected intraperitoneally and allowed to circulate for 2 min. A green laser light (532 nm, 1.5 mm in diameter, 20 mW power) was positioned 1 cm anterior, and ±1.5 cm lateral to bregma (in the hemisphere contralateral to the preferred hand). This positioning corresponded to the forelimb motor region in the mouse (Tennant et al., 2011) and was intended to specifically impair reaching and grasping ability. The laser was positioned 5 cm above the surface of the skull and activated for 13 min (Balbi et al., 2017). Sham mice received the laser illumination first, followed by the rose bengal injection after the laser was turned off. This order of events exposes the sham mice to exactly the same procedures as the stroke mice but does not result in any lesion. Afterwards, the scalp was sutured, bupivacaine was applied on the incision site as a topical analgesic, and mice regained mobility before being returned to their home cage. At 6- and 24-h following surgery, mice had bupivacaine reapplied to their incision site. Following the stroke surgery, animals were reintroduced to the HASRA for one week. The position of the seed arm was left unchanged from where it was before stroke. The stroke surgery did not result in any mortality.

### Assessment of lesion size and location

Following the conclusion of the experiment, mice were deeply anesthetized with sodium pentobarbital, decapitated, and their heads placed in 10% neutral buffered formalin at 4°C for four months. Brains were then removed and cryoprotected in 30% sucrose in PBS until saturated. Brains were then frozen at −80°C and every second coronal section (50 μm/section) from the olfactory bulb to the posterior portion of the hippocampus was mounted on gelatin-coated slides and stained with cresyl violet. Slides were scanned using a flatbed slide scanner (Canon 900F MKII) at a resolution of 1200 dpi. The area of damaged tissue was delineated on each section using ImageJ, with infarct volume calculated as: Σ(area of damage on each section) × tissue volume between each section (100 μm).

### Statistical analysis

All statistical analysis was performed using SPSS Statistics (v26, IBM Corp). In experiment 1, separate Kruskal–Wallis tests were used to assess task engagement on day 1 of training in each of the individual groups (low cycle, low single, high cycle, high single), and also collapsed across the arm height (low vs high) and presentation style (cycle vs single) variables. Total time spent in the task each day, daily qualitative score, and mean success rate were analyzed using repeated-measures ANOVA, with training day as a within-subjects factor and both arm height and presentation style as between-subjects factors. The Greenhouse–Geisser correction was applied to all repeated-measures analyses. *Post hoc* analysis was performed using Sidak-corrected *t* tests. Family-wise Type I error was controlled within each outcome measure to α = 0.05 as the significance cutoff. Proportion of each group that showed reaching at least once by a given day was analyzed using Log-rank (Mantel–Cox) χ^2^ survival tests. In experiment 2, total time spent in the task each day, daily qualitative score, and mean success rate were analyzed using the same repeated-measures ANOVA methodology as used for experiment 1.

## Results

### HASRA parameter optimization

#### Task engagement

**Figure 2.**
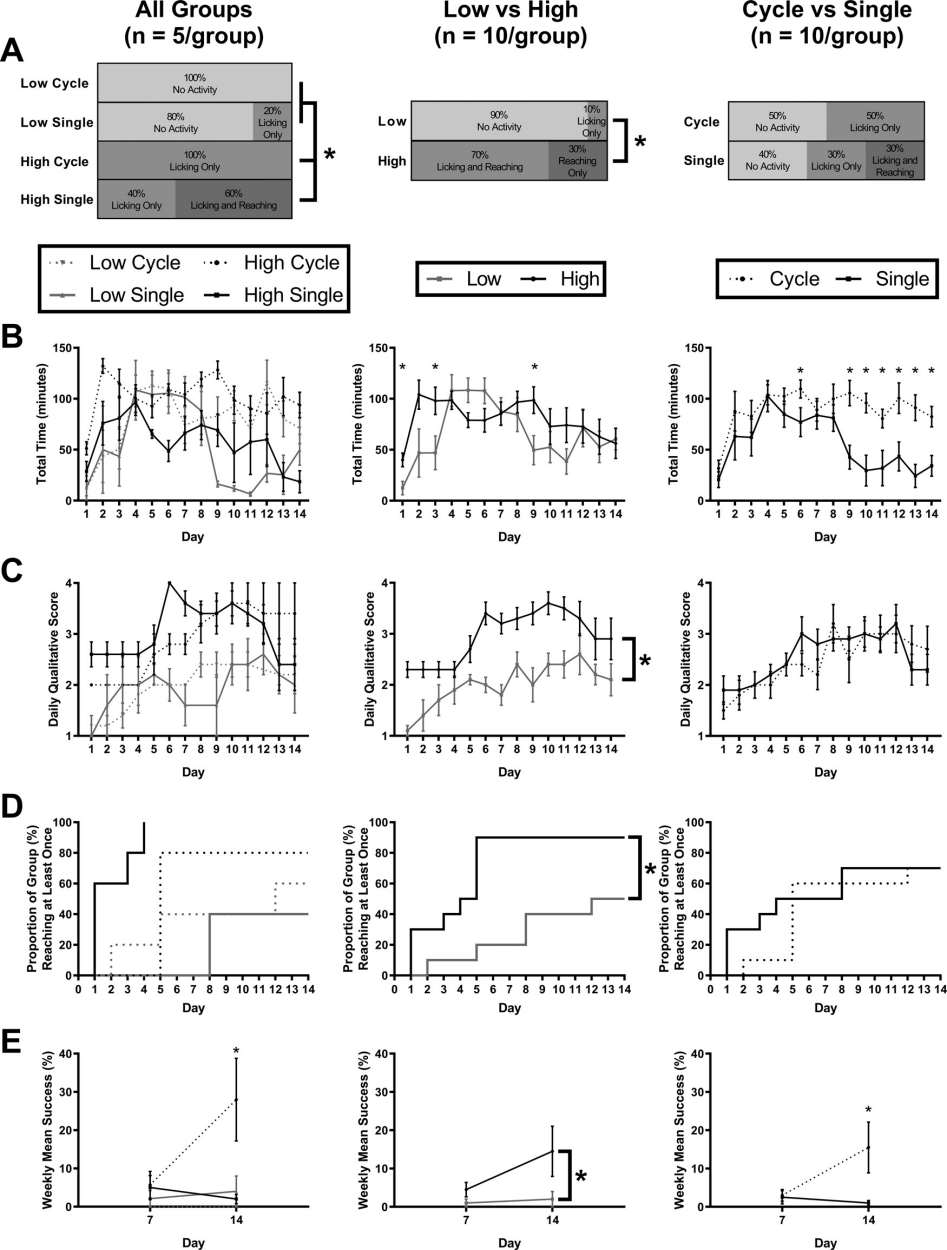
HASRA parameter optimization. ***A***, Proportion of each group showing varying behaviors on day 1 of exposure to the task. ***B***, Mean time per day (minutes) per mouse in which the pellet arm was delivering pellets for each group. This measure of time was used to avoid counting time in which the mouse rapidly entered and exited the HASRA without waiting to actually be delivered at least one pellet. Time in which the animal remained focused on the task long enough to be delivered a pellet more accurately represents, “task engagement.” ***C***, Mean daily qualitative score for each group based on scoring five videos per animal per day. ***D***, Survival curve of percentage of mice that displayed a qualitative score of at least 3 (reaching or licking) by day of training. ***E***, Percentage of successful reaching events based on scoring of 20 events per mouse at each time point. All data are represented as mean ± SEM; * represents a *post hoc* statistical effect of *p* < 0.05.

To assess the ability of the HASRA to initially engage animals to use the task, the daily qualitative score on day 1 was used as a metric of task engagement. A significant interaction between arm height and presentation style (*H*_(3)_ = 15.813, *p* = 0.001; Fig. 2*A*, left) indicated that the low cycle and low single groups had equivalently low levels of engagement (0% and 20%, respectively), while the high cycle and high single groups had significantly greater levels of early engagement (100% in both groups, with high single having 60% of mice both licking and reaching on day 1). Overall, arm height had a significant impact on task engagement (*H*_(1)_ = 14.646, *p* < 0.001; Fig. 2*A*, center), with 10% of animals with the low (floor height) arm, and 100% with the high (0.8 cm above floor) arm engaged in the task on day 1. Conversely, seed presentation style did not significantly impact day 1 engagement (*H*_(1)_ = 1.058, *p* = 0.304; Fig. 2*A*, right) with 50% engagement with a cycling style, and 60% engagement with a single presentation style.

The ability to not only engage animals early in training but also to sustain their interest in using the HASRA long-term (across weeks) was deemed essential. This was quantified using the total time each group spent using the task across days. No arm height by presentation style interaction was observed (*F*_(5.48,87.61)_ = 1.772, *p* = 0.121; Fig. 2*B*), but significant main effects of both arm height (*F*_(5.48,87.61)_ = 3.529, *p* = 0.005) and presentation style (*F*_(5.48,87.61)_ = 2.454, *p* = 0.035) were observed. *Post hoc* analysis of these effects indicated that the high arm height resulted in greater time-on-task on days 1, 3, and 9 (*p* < 0.05), whereas cycling presentation style resulted in greater time-on-task on days 6 and 9–14 (*p* < 0.05).

#### Adoption of reaching strategy

We sought to identify the HASRA parameters that would result in the greatest proportion of mice adopting reaching behavior (daily qualitative score ≥3) in the fewest number of days possible. There was a significant interaction among the time, arm height, and presentation style factors (*F*_(4.13,66.07)_ = 2.643, *p* = 0.040), with the mean qualitative score of the high arm across time being significantly greater than that of the low arm (*F*_(1,16)_ = 17.899, *p* = 0.001; Fig. 2*C*). In comparison, the single and cyclical presentation styles weren’t statistically different overall (*F*_(1,16)_ = 0.479, *p* = 0.499).

The day by which each group first displayed reaching behavior (daily qualitative score ≥3) was significantly different between the four groups (χ^2^_(3)_ = 21.38, *p* < 0.0001; Fig. 2*D*). The high presentation showed a significantly greater adoption of reaching behavior (90% of group by day 5) compared with the low presentation (50% of group by day 12, χ^2^_(1)_ = 7.752, *p* = 0.009). However, the single (70% by day 8) and cyclical (60% by day 5) presentations styles did not significantly differ (χ^2^_(1)_ = 0.298, *p* = 0.585) and achieved similar proportions of animals displaying reaching behavior (Fig. 2*D*). Overall, the high single group achieved both the greatest and most rapid adoption of reaching behavior, with 100% of the group having shown reaching behavior by day 4.

#### Reaching success rate

Success rate was a quantitative assessment of the percentage of successful reaches among the 20 events scored for each mouse every 7 d. A significant time by arm height by presentation style interaction (*F*_(1,16)_ = 7.750, *p* = 0.013) indicated that the high cycle group was the only one to significantly improve its reaching success rate by day 14 (Fig. 2*E*). Beneficial main effects of high arm height (*F*_(1,16)_ = 4.766, *p* = 0.044) and cyclical presentation style (*F*_(1,16)_ = 10.468, *p* = 0.005) were also observed. Overall, the high single group had displayed the greatest task engagement on day 1 and the most rapid adoption of reaching behavior. However, the significantly greater reaching success rate of the high cycle group at day 14 was deemed to be the most important element of task learning. Therefore, the high cycle parameters were selected for replication and testing for sensitivity to lesion-induced impairments in experiment 2.

### Distribution of events by circadian phase

We were also interested in when mice performed their reaching, and whether animals were relatively more successful at reaching during one phase of the light/dark cycle. In experiment 2, at day 22 (day before stroke), we manually scored all events from all animals (*N* = 5159 events). Mice were well trained by day 22; obtaining the seed by licking in 22% of the events (1159) and attempting to obtain the seed using their hand in 78% of events (4000). Of this subset of reaching events, 11.5% (462) were misses, 51.0% (2040) were knock-downs, and 37.5% (1498) were successful retrievals (Table 1).

**Table 1:** Total reaching event counts by phase of light cycle^a^

		Reaching event type			
Determinant	Total attempts	Miss	Knock-down	Success	*p* value
Light phase	715	*100 (14.0%)	*335 (46.8%)	280 (39.2%)	0.018
Dark phase	3285	*362 (11.0%)	*1705 (51.9%)	1218 (37.1%)	
Total	4000	462 (11.5%)	2040 (51.0%)	1498 (37.5%)	

a Total count of each type of reaching event on day 22 of training, split by phase of the light cycle. An attempt was any event where the hand passed through the front slot, resulting in either a miss, knock-down, or successful retrieval of the seed. The number in parentheses shows the percentage of that type of event within a given row. χ^2^ tests were used to assess differences in the relative distribution of event types across the light cycle (light phase vs dark phase). Cells prefixed by a * had significant differences in their relative distribution between light phases. Overall, 82.1% of reaching events occurred during the dark phase.

**Figure 3.**
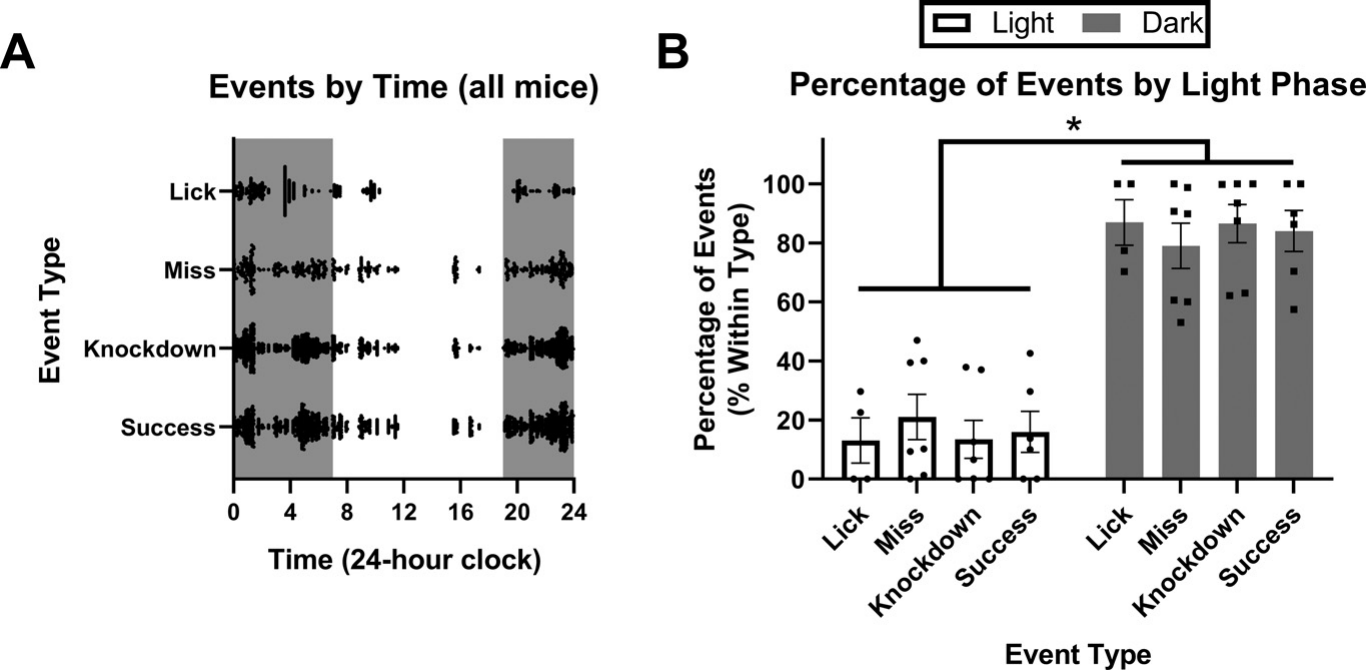
Distribution of events by circadian phase. ***A***, Plot of all events for all mice on day 22 (day before stroke) of experiment 2. Each event of a given type is represented by a single black dot. The white portion of each graph represents the light phase of the light cycle (7 A.M. to 7 P.M.), whereas data in the gray portion of the graph represent data from the dark phase (7 P.M. to 7 A.M.). ***B***, Mean percentage of each event type by portion of the light phase. Mean for individual mice are represented by single dots. *N* = 5159 events, *n* = 7 mice. All data are represented as mean ± SEM; * represents a *post hoc* statistical effect of *p* < 0.05.

Mice displayed continuous activity in the HASRA during the dark phase, whereas during the light phase mice displayed a period of complete inactivity from ∼12 to 3 P.M. (Fig. 3*A*). Overall, mice performed significantly more of all types of events in the dark phase than in the light phase (lick, *t*_(3)_ = −4.808, *p* = 0.012; miss, *t*_(6)_ = −3.787, *p* = 0.009; knock-down, *t*_(6)_ = −5.681, *p* = 0.001; success, *t*_(5)_ = −4.900, *p* = 0.004; Fig. 3*B*). In both phases, the relative percentage of successful attempts did not significantly differ; however, misses were significantly more frequent during the light phase, whereas knock-downs happened significantly more frequently during the dark phase (χ^2^_(3)_ = 8.089, *p* = 0.018; Table 1).

### HASRA sensitivity to lesion-induced impairments

**Figure 4.**
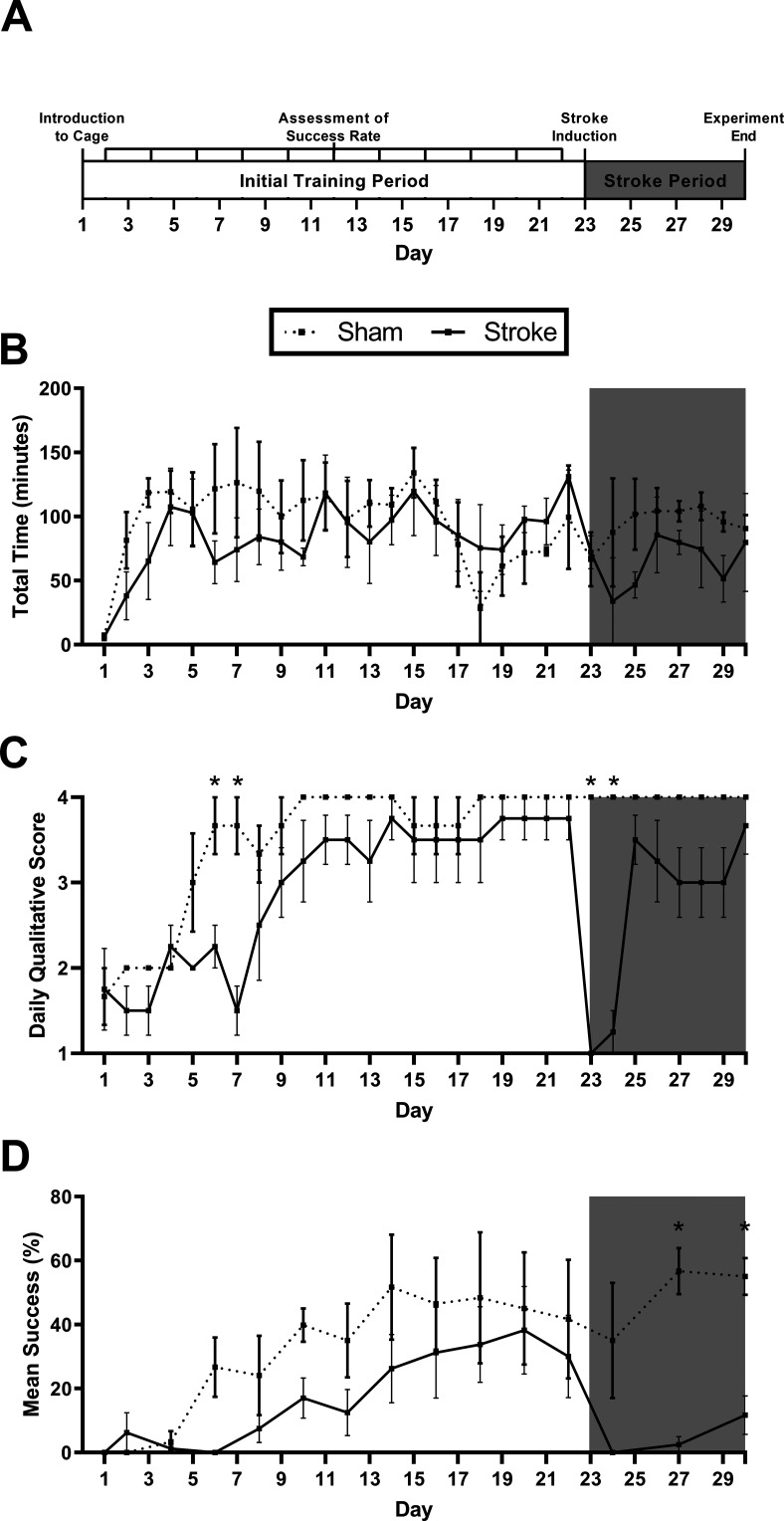
HASRA sensitivity to lesion-induced impairments. ***A***, Timeline of experiment 2. ***B***, Mean time per day (minutes) per mouse with the pellet arm activated for each group. ***C***, Mean daily qualitative score for each group based on scoring five videos per animal per day. ***D***, Percentage of successful reaching events based on scoring of 20 events per mouse at each time point. The white portion of each graph represents time points before stroke, whereas data in the gray portion of the graph represent poststroke time points. All data are represented as mean ± SEM. *N* = 7 for all panels; * represents a *post hoc* statistical effect of *p* < 0.05.

In experiment 2, mice received 22 d of exposure to the HASRA before receiving a photothrombotic stroke (or sham surgery on day 23) and seven additional days of testing. Mice had their qualitative score assessed daily throughout this period, and their success rate (based on 20 events) scored every second day (Fig. 4*A*). No significant differences in time-on-task were observed between the sham and stroke groups, neither before, nor after surgery (*F*_(2.05,10.24)_ = 1.184, *p* = 0.346; Fig. 4*B*). Despite suffering a stroke, mice were still able to engage with the HASRA. Daily qualitative scoring indicated that the stroke group performed significantly worse than the shams on days 6 and 7 of training, but then caught up by day 23 when stroke occurred. Stroke resulted in a drastic reduction in qualitative performance on the day-of, and day-after stroke, that then rapidly recovered (*F*_(3.13,12.51)_ = 5.951, *p* = 0.009; Fig. 4*C*). On these days, despite spending significant amounts of time in the HASRA, stroke mice were unable to recover seeds, even by licking. Finally, success rates were not significantly different between groups before stroke; however, stroke animals displayed a significant decrease in performance at all points following stroke, and were significantly impaired relative to shams at days 27 and 29 (*F*_(13,52)_ = 2.721, *p* = 0.005; Fig. 4*D*).

### Verification of animal health and lesion induction

**Figure 5.**
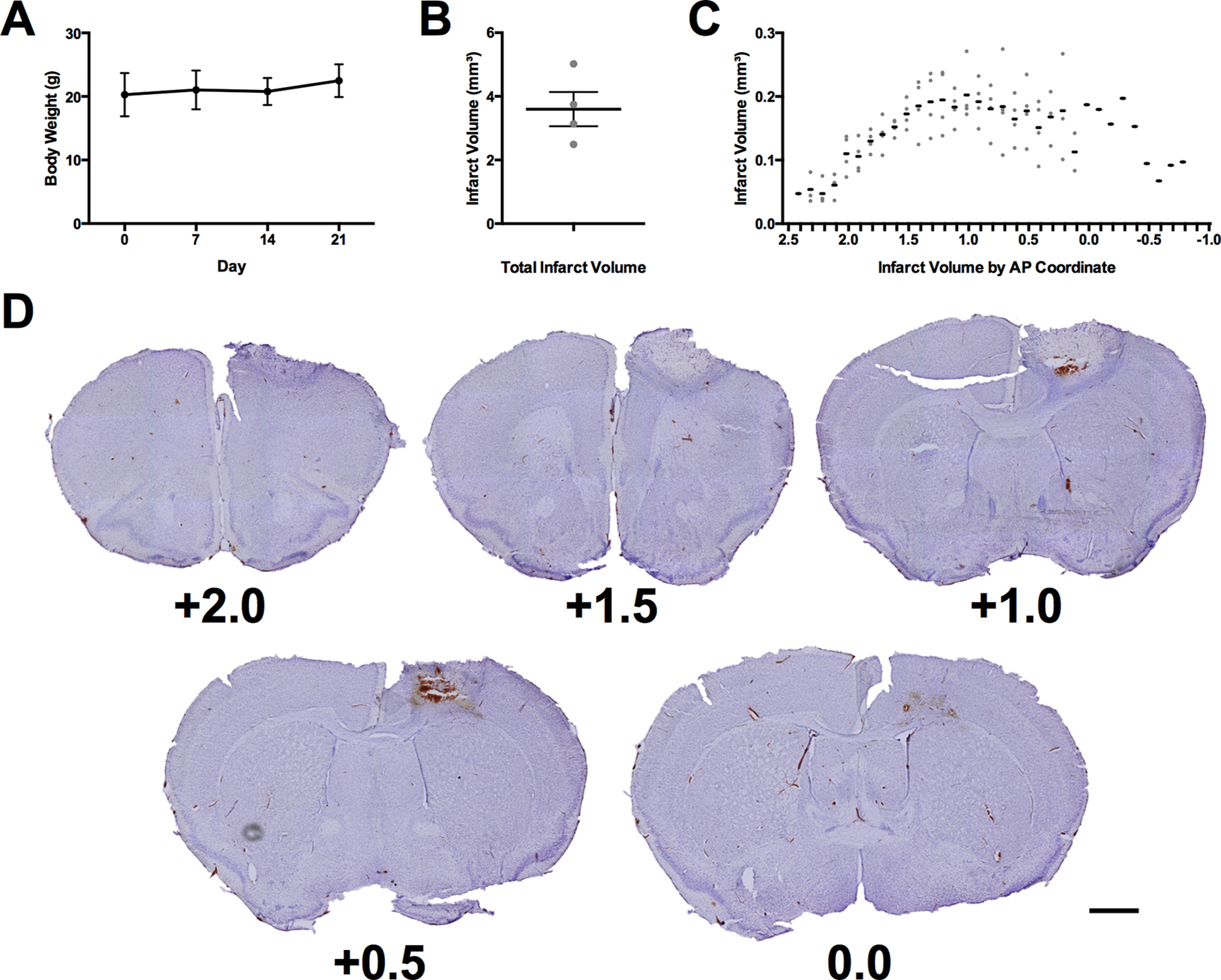
Verification of animal health and lesion induction. ***A***, Mean body weight of all mice throughout experiment 2. ***B***, Mean total infarct volume of all mice receiving stroke. ***C***, Infarct volume of all mice receiving stroke at each anteroposterior coordinate of damage relative to bregma. ***D***, Representative cresyl violet images of lesion in the mouse with the total infarct volume closest to the mean (3.75 mm^3^). Each image is labeled with its distance anterior to bregma in millimeters. Scale bar = 1 mm. The black lines in panels ***A–C*** represent the group mean ± SEM. The gray dots in panels ***B***, ***C*** represent the infarct volumes of individual animals. *N* = 7 for panel ***A*** and *n* = 4 in panels ***B***, ***C***.

Throughout experiment 2, body weights were monitored to ensure that no significant reduction in body weight occurred because of food restriction (Fig. 5*A*). All animals maintained healthy body weights with no adjustment in food ration required. Photothrombotic stroke resulted in a mean lesion volume of 3.59 ± 0.54 mm^3^ (Fig. 5*B*) with a maximum extent from +2.4 mm anterior to −0.8 mm posterior to bregma (Fig. 5*C*). These lesions were centered in the region comprising the forelimb motor region of the mouse (Fig. 5*D*; Tennant et al., 2011).

## Discussion

We developed an automated system for delivering single pellet reach training in a home-cage housing environment and validated the efficacy of this device in two experiments. In experiment 1, we demonstrated that delivering seeds 0.8 cm above the tube floor (at approximately mouth level) using a cyclical pattern (every 5 s) resulted in the greatest reaching success rate after two weeks of training. This target height is similar to that used in the classic single-pellet reaching task (Farr and Whishaw, 2002) and resulted in 100% of mice engaging with the task and obtaining seeds on the first day of exposure to the HASRA. This is notable, as it demonstrates that the HASRA rapidly engages mice in the task even without prior habituation to the millet seeds or caging environment. 80% of mice that used these “high cycle” device parameters showed reaching behavior by day 5 of training and this subset of mice attained a reaching success rate of ∼35% by day 14 of training.

In experiment 2, we demonstrated the sensitivity of the HASRA to detect lesion-induced impairments. We used the optimized, high cycle parameters in a new group of mice and found that 100% of mice displayed reaching behavior by day 9 of training. By day 14, mice had once again attained a reaching success rate of ∼35% and allowing additional training to day 22 did not significantly improve this performance (∼37%). Following stroke, mice continued to spend a significant period of time in the tube but did not attempt to use their hands to reach for the first 2 d following the surgery. By day 7 poststroke, two of the four mice (50%) with stroke were able to show some reaching success, but this was still significantly reduced compared with shams and their own pre-stroke performance (∼17.5% success). Overall, this level of reaching success and stroke-induced impairment is comparable to that observed in the single-pellet reaching task (Farr and Whishaw, 2002; Chen et al., 2014).

The HASRA advances previous efforts to automate single-pellet reaching (Torres-Espín et al., 2018) by integrating a robotic pellet dispenser directly within the animals’ home-cage environment. This has the benefit of reducing the potential for experimenter-induced stress (Balcombe et al., 2004) and allows the mice to perform the task at any phase of the light-cycle *ad libitum*. As has been previously observed (Silasi et al., 2018), we found that mice were most inclined to reach during the dark phase, but this is at odds with the majority of behavioral testing paradigms where the experimenter conducts testing during the light phase as a part of their workday. The limited time available to an individual experimenter constrains the amount of training that can be delivered to animals in one-on-one training sessions. By using the HASRA, mice were able to each receive 100+ min of reaching practice per day, with the total number of mice that could be trained limited only by the number of cages deployed. This high-intensity training was accompanied by individualized training progression for each mouse using a combination of RFID tagging and motorized stages to customize pellet positioning and optimally shape reaching behavior. Each HASRA unit is capable of delivering consistent, yet individualized, training conditions to each animal, potentially reducing the training variability that may be observed with human experimenters (Fouad et al., 2013).

Future development of the HASRA could focus on presenting other types of items, such as traditional reaching pellets, and automating the quantification of mouse performance within the device. Recent developments in markerless tracking methods (Mathis et al., 2018) could enable kinematic analysis of reaching behavior with the use of a high frame-rate camera. Furthermore, convolutional neural nets could be used to automate trial classification (Ryait et al., 2019), which was performed manually in the present study. The current design already employed an image classification scheme to determine when new pellets should be presented during the “single presentation” style in experiment 1. Automated classification of trial outcome would make it feasible to obtain detailed data on every reaching event performed by each mouse, which is beyond the scope of what can be performed by human experimenters given the thousands of reaching events that mice can perform within a single day.

Overall, the HASRA provides novel automation of the classic single-pellet reaching task within a home-cage environment. This task can be used to facilitate motor training experiments on a massive scale that has previously been impossible because of the demands of one-on-one behavioral testing.

## Synthesis

Reviewing Editor: Sunghee Cho, Burke Neurological Institute/Feil Family Brain and Mind Research Institute, Weill Cornell Medicine Decisions are customarily a result of the Reviewing Editor and the peer reviewers coming together and discussing their recommendations until a consensus is reached. When revisions are invited, a fact-based synthesis statement explaining their decision and outlining what is needed to prepare a revision will be listed below. The following reviewer(s) agreed to reveal their identity: Gordon Teskey.

The presented study, describing a detailed construction and usage of an automated device for a skilled reaching test in an experimental animal model of stroke, were evaluated by two reviewers who have expertise in the field. Both reviewers considered the automated device is a clever and improved addition to the field gauging functional recovery. They expressed strong enthusiasm of the use of device and acknowledged the advantage for its ability to self-train in its task, which requires substantial effort and. There are however several concerns. These include the extent of food reduction that may affect other aspects of behaviors, clarification on how frame variation is conducted, and the absence of histological data to show the severity of stroke. More detailed and specific comments from reviewers are below.

Reviewer 1

This paper presents the construction and usage of an automated device to train and assess the performance of mice on a single item skilled reaching task in their home cage under group housed conditions. This is important because the use of a high-quality and reliable automatic device can reduce labor intensive training and assessment as well as reduce experimenter-induced variability and the stress associated with human contact.

The paper does an excellent job of providing the necessary materials to construct and operate the automatic device. They also compared mouse learning performance with two different pedestal heights (as well as proximal/distal and medial/lateral) and at two different inter-item intervals. Moreover, they assessed the performance at different phases during the light-dark cycle and following a photothromic stroke to motor cortex.

Major

The authors have made a serious attempt to reduce murine stress levels and provide a more naturalistic context in which to train and assess skilled reaching behaviour in group housed mice. But did this new context also alter mouse behaviour? How did they restrict mouse chow to 1g/mouse/day in group housed mice? Did some mice guard the entrance to the reaching tube and obtain much more than 20 millet seeds? Could they be stopped from obtaining more pellets? Were there agonistic encounters between mice to enter the reaching tube? How much of each animal’s daily diet (in g/mouse/day) was composed of millet seeds and what was the range across the mice?

In the “Video recording and seed detection” section there is clarification required for the following sentences, “These frames were manually classified by an experimenter and subjected to random variations to increase the generalizability of conditions under which the detection would properly operate. These frame variations included vertical flipping, random 126 rotation, zoom in/out, brightness adjustment, and width/height shifting. The training set contained 90% of 127 frames (n = 4934) and was trained for 50 epochs with a batch size of 32. We utilized Adam as optimizer and Binary cross-entropy as loss functions. Only the weights with the lowest validating loss were preserved.” Please clarify how the frame variations were conducted? What is “Adam”? What is Binary cross-entropy? How were weights with the lowest validating loss preserved”.

That authors state that maintenance was performed when successful presentation dropped below 90%. How often did this occur? What is the failure rate of the apparatus? Where there other engineering issues with the construction and maintenance of the device? How long do the devices last?

Minor

Consider altering the title to “... the single item reaching task.” since the study used a millet seed rather than the traditional pellet.

Consider replacing the word “paw” with “hand”.

Provide a citation for the photothrombic stroke method.

The day/night phase experiment should be presented before stroke data.

What other types of food could be used effectively - pellets?

What was the 65% reaching failure rate due to and is this the same or different from traditional methods of assessing reaching success?

Does the successful use of the apparatus generalize to other mouse strains?

Reviewer 2

Here the authors present a new device that enables group housed mice to self-train in a skilled reaching task from their home cage. Radio frequency tagging permits task parameters to be tailored per individual mouse and event triggered video recordings to be linked with them. The description of the task elements is nicely done and it has numerous clever elements. Experiment 1 established strategies for pellet delivery that best encouraged reaching, which was then applied to characterize learning curves and test the effects of motor cortical infarcts on performance in Experiment 2. There are two major issues that partially detract from confidence in the potential of this automatic task, at least one of which may require new data to address. The other issues are more minor and probably remedied with minor clarifications and revisions.

Major:

1. Mice underwent rather extreme food deprivation (1g/day). This is less than half of the typical ad lib feeding in adults (∼ 3g, more or less depending on age and sex). Assuming this isn’t a typo, is this really necessary to motivate performance? It is definitely not necessary in traditional reaching tasks. If the automated task does require this level of food deprivation, it would limit its usefulness to studies that have no reason for concern about the brain and behavioral effects of major food restriction (which ≠ stroke models).

That mouse weights are not reported does not help this issue. Were weights were monitored over the course of the experiments? If so, they should be reported.

2. There is no histological characterization of the photothrombotic infarct extents. This limits the capacity to gauge whether the injuries would be likely to yield chronic impairments in traditional reaching tasks. Without this, minus direct comparison with an established task (which I am NOT suggesting), this leaves opens the question of how sensitive the approach may be relative to traditional reaching tasks for detecting chronic forelimb impairment, i.e., it fails to compel its usefulness for stroke recovery studies.

If the brains are not available from the mice that were used in this study, the next best option may be to reproduce the infarcts as closely as possible in a separate set of mice for histological characterization.

Less Major:

The daily task time per mouse is rather variable (Fig. 3B). Does this reflect mostly day-to-day variance within individual mice? Or did some mice consistently spend less time than others in the device? If the latter, the authors should consider how social hierarchies might contribute to that variance, especially in the males (e.g., Beery et al., 2020, doi: 10.1016/j.neubiorev.2020.03.033)

Minor:

- The reaching task that is being automated is known as the “Single Seed Reaching Task” (Xu et al., 2009, Nature, doi:10.1038/nature08389). It is fine with this reviewer if the authors instead refer to it as a single pellet task, but they should make clear in the Intro that their meaning of this includes the single seed variation.

- It is not mentioned until deep in the methods (p. 8) that the automated task permits control of the reaching side (left vs. right forelimb). Because the ability to control that is crucial for many applications, the authors should consider making a much more prominent point of this capacity, e.g., in the Abstract. It also doesn’t hurt to highlight it given that the strategy for controlling it is quite clever.

- The meaning of active arm time needs clarification. Does this mean the time during which the arm was presenting a pellet? It would also be appropriately to more prominently consider the extent to which this reflects task engagement.

- What age were the mice?

- The three “Training stages” are presented followed by explanation of how they are being controlled (“Daily qualitative scoring”). To avoid leaving the reader to wonder about the latter while reading the former, the authors might either flip the order of these sections or mention early in the “Training stages” section that the transitions between them were controlled by an experimenter based on video monitoring.

- What was the rationale for the specific locus of the photothrombotic infarcts? Where within motor cortex the infarcts were centered should be mentioned (since “motor cortex” is a big place).

- In the Introduction: “individual differences between experimenters has a significant effect on the quality of training and overall reaching success rate that is achieved”. This phrasing needs to be adjusted to suggest the effect as a possibility rather than a given, since other studies have failed to detect significant experimenter effects.

- In the following sentence, “...when this criterion is met significant differences can be observed across groups of subjects.”. If the authors are referring to animal batch effects here, this is of unclear relevance to the focus since removal of experimenter contributed variance doesn’t prevent batch effects.

- The authors might tone down statements regarding experimenter time costs of traditional reaching tasks (∼10 min/day/mouse). A bigger advantage of the automated task could be that it increases the amount of time that mice are engaged in the task per day.

## Author Response

Response to Editor:

Dear Dr. Cho,

We thank you and the reviewers for your consideration of our manuscript (eN-MNT-0242-20) and would like to extend our appreciation for the thoughtful and constructive feedback provided by both reviewers. Both reviewers expressed great interest in the HASRA, describing it as, “nicely done", with “numerous clever elements", and that the associated manuscript, “does an excellent job providing the necessary materials to construct and operate the automatic device.”

Each reviewer provided invaluable commentary on the report, which has allowed us to clarify our findings and improve the quality of the manuscript even further. Primarily, both reviewers expressed concern regarding the food restriction paradigm and lack of infarct volumes in the original manuscript. We have provided additional sections and figure panels in the manuscript to thoroughly address these concerns, while also performing supplementary analyses of our data to be able to fully answer all queries that were raised. Our response to each comment can be found below, point-by-point, and all changes to manuscript itself have been made in bold and red font. We hope that you will consider this revised version for publication in eNeuro.

---------------------------------------------

Synthesis of Reviews:

Synthesis Statement for Author (Required):

The presented study, describing a detailed construction and usage of an automated device for a skilled reaching test in an experimental animal model of stroke, were evaluated by two reviewers who have expertise in the field. Both reviewers considered the automated device is a clever and improved addition to the field gauging functional recovery. They expressed strong enthusiasm of the use of device and acknowledged the advantage for its ability to self-train in its task, which requires substantial effort and. There are however several concerns. These include the extent of food reduction that may affect other aspects of behaviors, clarification on how frame variation is conducted, and the absence of histological data to show the severity of stroke. More detailed and specific comments from reviewers are below.

Reviewer 1

This paper presents the construction and usage of an automated device to train and assess the performance of mice on a single item skilled reaching task in their home cage under group housed conditions. This is important because the use of a high-quality and reliable automatic device can reduce labor intensive training and assessment as well as reduce experimenter-induced variability and the stress associated with human contact.

The paper does an excellent job of providing the necessary materials to construct and operate the automatic device. They also compared mouse learning performance with two different pedestal heights (as well as proximal/distal and medial/lateral) and at two different inter-item intervals. Moreover, they assessed the performance at different phases during the light-dark cycle and following a photothromic stroke to motor cortex.

Major

- The authors have made a serious attempt to reduce murine stress levels and provide a more naturalistic context in which to train and assess skilled reaching behaviour in group housed mice. But did this new context also alter mouse behaviour?

Response: We did not observe any overt changes in the animals’ behaviour upon first introduction to the HASRA. Over time, we observed that some mice began to exhibit occasional chewing of the front wall around the reaching slot; however, in subsequent studies we have found that this can be discouraged by replacement of the acrylic front wall with a metallic material. Despite the chewing behaviour, mice continued to exhibit reaching success. We have added additional details to the “Video recording and seed detection” section regarding this occasional chewing behaviour.

- How did they restrict mouse chow to 1g/mouse/day in group housed mice?

Response: The reviewer is correct that we were not able to monitor or control individual food consumption in group housed mice. Our restriction protocol was to provide 1g of chow per mouse/day. For example a cage of 5 mice received 5g of food each day. Importantly, we made efforts to distribute the food evenly among mice by splitting the chow into small pieces and spreading it throughout the cage, thus allowing all animals a chance to forage and obtain it. Upon adding food to the cage, we often observed all mice beginning to immediately forage and eat the food. Details regarding this food administration procedure have been added to the “Validation experiment design” section.

- Did some mice guard the entrance to the reaching tube and obtain much more than 20 millet seeds? Could they be stopped from obtaining more pellets? Were there agonistic encounters between mice to enter the reaching tube?

Response: We did not observe mice guarding the entrance to the reaching tube or any aggressive encounters between mice attempting to enter the reaching tube. The majority of mouse time when not in the tube seemed to be spent nesting together in a cardboard shelter that was placed within each mouse cage as part of standard caging procedures. Overall, having ad libitum access to the HASRA seemed to avoid any aggression associated with its use. In experiment 2 (with stroke) the mean time spent in the HASRA (averaged for each animal across all days in the study) ranged from 55 to 114 minutes per day. With a new pellet delivered every 7 seconds, this equates to the animal with the least exposure to the device still having nearly 500 opportunities to obtain pellets each day. The design of the apparatus allows programmatic control over how many trials a mouse could engage in per day; however, for the present study we wished to quantify how mice would naturally engage with the HASRA.

- How much of each animal’s daily diet (in g/mouse/day) was composed of millet seeds and what was the range across the mice?

Response: We did not measure the amount of food individually consumed by each mouse, nor the total number of successful pellet retrievals by each mouse each day (only a sample of events was scored). However, an estimate of daily millet seed consumption can be obtained from Experiment 2, day 22, where we scored all events for each mouse. Out of all mice that showed reaching success, mice obtained between 216 - 386 millet seeds on that day. Each millet seed weighs ∼6.4 mg, therefore, our mice consumed between 1.38 and 2.47 grams of millet seeds on day 22. This likely composes more than half of their daily food intake considering the 1 g/mouse/day ration of regular chow that they are also provided.

- In the “Video recording and seed detection” section there is clarification required for the following sentences, “These frames were manually classified by an experimenter and subjected to random variations to increase the generalizability of conditions under which the detection would properly operate. These frame variations included vertical flipping, random 126 rotation, zoom in/out, brightness adjustment, and width/height shifting. The training set contained 90% of 127 frames (n = 4934) and was trained for 50 epochs with a batch size of 32. We utilized Adam as optimizer and Binary cross-entropy as loss functions. Only the weights with the lowest validating loss were preserved.” Please clarify how the frame variations were conducted? What is “Adam”? What is Binary cross-entropy? How were weights with the lowest validating loss preserved”.

Response: We have completely revised the paragraph in question, making an effort to provide only the high-level “what and why” of the pellet detection system, rather than the exact details on “how” as we feel that this is outside the scope of the paper. The goal of this paragraph is to make our pellet detection system accessible to readers with only a basic understanding of neural networks. More details on the exact implementation of our frame adjustments and network training are provided on the GitHub for this project. For the purpose of answering the reviewer’s queries, the details available in the GitHub are provided as follows:

1. The five following random operations composed the frame variations. These operations occurred during the training process.

1.1. Random vertical flipping

Firstly, a random number is generated under continuous uniform distribution from 0 to 1. If the value is greater than 0.5, then a vertical flipping is applied to the frame. Otherwise, the frame is kept as the same.

1.2. Zoom in/out

The transformation rate was randomly generated from 0.8 to 1.2 which following uniform distribution. Each frame was randomly resized and cropped if zoom in or padded by zeros if zoom out.

1.3. Brightness adjustment

The brightness adjustment rate was randomly generated under uniform distribution in the range from 0.5 to 1.2. The brightness of the frame was altered by this rate.

1.4. Width/height shifting

For the width shifting, the frame was shifted by a number randomly generated by uniform distribution from -10% to 10%, (10% left shifted to 10% right shifted). Height shifting was either 10% upward to 10% downward.

1.5. Rotation

A rotation angle was generated under uniform distribution from -5 degrees to 5 degrees. Then the rotation was applied to the frame.

2. Adam optimization algorithm

Please find details about this algorithm in the original paper, Kingma and Ba. (2014). Adam: A Method for Stochastic Optimization. arXiv, 1412.6980.

Adam is an optimization algorithm with an adaptive learning rate. The whole algorithm can be briefly illustrated by the following formula:

While and are initialed as 0, are both hyper parameters. is the gradient of the neural network.

3. Binary cross entropy

Overall, binary cross entropy is a kind of Kullback-Leibler divergence which measures the difference between two distributions. And we would like to shrink the gap between read data and the predictions of the neural network. That is reason here why we are using binary cross entropy to evaluate our model.

Here is the formula:

Y is the true label which can only be 0 or 1. p(y) is the probability of y, which can be regarded as the prediction of the neural network. indicates which sample in a mini batch. And N means the total number of samples in the batch.

We have removed any mention of these optimizer and loss functions from the manuscript, as we feel that these low-level details are not directly relevant to the purpose of the paper and are fairly complex components that would take too much space to elaborate on. This information will be provided in the GitHub, which will be more relevant to those seeking to actually implement the software and pellet detection algorithm.

- That authors state that maintenance was performed when successful presentation dropped below 90%. How often did this occur? What is the failure rate of the apparatus? Where there other engineering issues with the construction and maintenance of the device? How long do the devices last?

Response: A new paragraph has been added to the “Video recording and seed detection” section of the methods to elaborate on regular maintenance required for the HASRA. Pellet detection for the purpose of signalling a need for maintenance was implemented in Experiment 2. The successful presentation rate was checked every morning and maintained above 90%. One of the cages dropped below this threshold in successful presentation at days 7, 13, and 15. The other cage stayed above this threshold on all days. If this need for maintenance is defined as the “failure rate” of the apparatus, then this would indicate a failure rate of 10% in one cage and 0% in the other across the 30-day period of Experiment 2. Multiple strategies were used to increase successful presentations of the HASRA back over the 90% threshold: filling up the hopper with seeds, removing crumbs from the ∼1mm deep well at the tip of the motorized arm and bottom of the hopper using a brush, and completely replacing the arm with a new one. However, it should be noted that none of these maintenance activities required the HASRA to be taken offline for more than a few minutes. No other mechanical issues were reported during these experiments.

We should note that the components used in this version of the HASRA were also used in previous beta versions of the device and have continued to be used in subsequent versions. We have not quantified or tracked the lifespan of any of the components; however, it is evident that it is possible for many components of the device to function through months of continual usage without failure.

Minor

- Consider altering the title to “... the single item reaching task.” since the study used a millet seed rather than the traditional pellet.

Response: We understand the reason for making this suggestion, however our apparatus was designed to automate a very well designed and characterized task: the single pellet reaching task. Re-naming our task a “single item task” may lead to confusion as it has so many features in common with the single pellet task. Indeed, other published papers using the manual version of the single pellet task often use millet seeds as “pellets”. See “Chen CC, Gilmore A, Zuo Y. Study motor skill learning by single-pellet reaching tasks in mice. J Vis Exp. 2014;(85):51238. Published 2014 Mar 4. doi:10.3791/51238"

- Consider replacing the word “paw” with “hand”.

Response: Thank you for pointing out this oversight on our part, the word “paw” has now been replaced with “hand” throughout the manuscript.

- Provide a citation for the photothrombic stroke method.

Response: A citation using the same photothrombotic stroke induction parameters as we did in this study has been added to the “Photothrombotic stroke induction” section.

- The day/night phase experiment should be presented before stroke data.

Response: The “Distribution of events by circadian phase” section has been moved before the “HASRA sensitivity to lesion-induced impairments section” and the figure numbers amended accordingly.

- What other types of food could be used effectively - pellets?

Response: The item presentation arm could be redesigned to accommodate a variety of different sized objects. Currently we have only gathered data on training the mice with millet seeds. However, in future iterations we would like to try using small nutritionally balanced food pellets to reduce any concerns surrounding food restriction. We have added a comment on this to the “future development” section of the Discussion.

- What was the 65% reaching failure rate due to and is this the same or different from traditional methods of assessing reaching success?

Response: As outlined in Table 1, failed reaches were complete “misses” in a minority of events (11.5%), “knockdowns” in the majority of events (51.0%), and successes 37.5% of the time. Misses could be comprised of any type of movement through the reaching slot that did not result in the pellet being disturbed from the tip of the arm, such as completely failing to contact the pellet, or touching the pellet with the digits, but not displacing it from the pellet arm. A knockdown could be comprised of an event such as hitting the pellet off of the arm or partially grasping the seed but dropping it before successful retrieval to the mouth. However, we did not make these subtle distinctions during our data collection and thus cannot elucidate the cause of reaching failure in more detail than currently provided in Table 1. The 37.5% reaching success rate that we obtained is comparable to that obtained by others using the more traditional single pellet task (Chen et al, 2014). This additional reference has been added to our Discussion when comparing the performance we obtained to the traditional single-pellet task.

- Does the successful use of the apparatus generalize to other mouse strains?

Response: In the current study, we only used a single strain of mouse (Thy1-ChR2-YFP) which was generated from a C57BL/6 x SJL hybrid that was backcrossed to C57BL/6. Previous studies have demonstrated rat strain differences in reaching success on the single pellet reaching task (VandenBerg et al., 2002). However, rodents are generally quite adept at reaching and grasping, and a wide variety of mouse strains have been demonstrated the ability to perform tasks within this motor domain (Tucci et al., 2007). Furthermore, other strains of mice have been shown to enter and interact with home cage forelimb assessment devices (Woodard et al., 2017). Therefore, although we have currently only tested a single strain of mouse in this study, we have no reason to believe based on the literature that HASRA would not be generalizable to other mouse strains.

Reviewer 2

Here the authors present a new device that enables group housed mice to self-train in a skilled reaching task from their home cage. Radio frequency tagging permits task parameters to be tailored per individual mouse and event triggered video recordings to be linked with them. The description of the task elements is nicely done and it has numerous clever elements. Experiment 1 established strategies for pellet delivery that best encouraged reaching, which was then applied to characterize learning curves and test the effects of motor cortical infarcts on performance in Experiment 2. There are two major issues that partially detract from confidence in the potential of this automatic task, at least one of which may require new data to address. The other issues are more minor and probably remedied with minor clarifications and revisions.

Major:

1. Mice underwent rather extreme food deprivation (1g/day). This is less than half of the typical ad lib feeding in adults (∼ 3g, more or less depending on age and sex). Assuming this isn’t a typo, is this really necessary to motivate performance? It is definitely not necessary in traditional reaching tasks. If the automated task does require this level of food deprivation, it would limit its usefulness to studies that have no reason for concern about the brain and behavioral effects of major food restriction (which ≠ stroke models).

Response: Food restriction is a commonly used element of the single pellet reaching task in both rats (Metz and Whishaw, 2000) and mice (Farr and Whishaw, 2002) in order to motivate task engagement. In the present study we adopted the food restriction guidelines that have been put in place for the single pellet reaching task within our institution. We agree with the reviewer that it may be possible that mice could still learn the task with a lesser degree of food restriction, or perhaps no food restriction once initial learning of the task has been completed. Utilizing the least food restriction possible would be ideal; however, the detailed experiments required to optimally titrate the food restriction for task learning were outside the scope of the present study.

In our response to a similar concern by Reviewer 1 we demonstrate that mice were likely still able to consume a typical ad libitum feeding amount (∼2-3 grams) by supplementing their 1 g/mouse/day ration with an additional 1.38 to 2.47 grams of millet seeds by using the HASRA. We have also added body weight data to a new Figure 5A, showing that the mice did not lose weight relative to their pre-restriction baseline throughout the experiment, and in fact slightly increased their weight. This is well within the weight restriction guidelines set by our institution (80% of free-feeding weight maximum reduction) and other single pellet reaching papers which commonly use 90% of free-feeding baseline as a target weight.

That mouse weights are not reported does not help this issue. Were weights were monitored over the course of the experiments? If so, they should be reported.

Response: We have added body weight data for Experiment 2 to Figure 5A. We have clarified in the “Validation experiment design” section that animals were weighed weekly. Individual animal weights varied between 88% to 122%, with a mean of 102% of free-feeding weight at these measurement points, which was within the maximum allowable reduction of 80% as defined by our institutional animal care committee. This indicates that despite the 1g/day restriction of regular food, all mice were able to supplement their food intake enough by using the HASRA to maintain an acceptable body weight. We have also reported the animal body weights in a new section, “Verification of animal health and lesion induction”.

2. There is no histological characterization of the photothrombotic infarct extents. This limits the capacity to gauge whether the injuries would be likely to yield chronic impairments in traditional reaching tasks. Without this, minus direct comparison with an established task (which I am NOT suggesting), this leaves opens the question of how sensitive the approach may be relative to traditional reaching tasks for detecting chronic forelimb impairment, i.e., it fails to compel its usefulness for stroke recovery studies.

If the brains are not available from the mice that were used in this study, the next best option may be to reproduce the infarcts as closely as possible in a separate set of mice for histological characterization.

Response: We have added a new methods section, “Assessment of lesion size and location", a new results section, “Verification of animal health and lesion induction", and a new Figure 5 (and associated caption), which shows the total infarct volume, distribution of the stroke volume across the anteroposterior axis of the brain, as well as a set of representative stroke images from the mouse with the stroke closest to the group mean. Overall, mean photothrombotic stroke volume was 3.6 mm3, which is similar to that produced by others (Bell et al., 2015; ∼2.8 mm3) in experiments of mouse post-stroke impairment/rehabilitation. In this study, Bell et al., demonstrate that their lesions also result in impairments in another skilled forelimb reaching/grasping task (pasta matrix reaching task), which can be recovered via high intensity reach training. The similar lesion size in the present study, and lesion-induced impairments in the HASRA suggest that our task may also be useful for studying the effects of high-intensity limb training. Tasks such as pasta matrix reaching are highly labour intensive, which is a limitation that the HASRA is uniquely situated to ameliorate.

Less Major:

The daily task time per mouse is rather variable (Fig. 3B). Does this reflect mostly day-to-day variance within individual mice? Or did some mice consistently spend less time than others in the device? If the latter, the authors should consider how social hierarchies might contribute to that variance, especially in the males (e.g., Beery et al., 2020, doi: 10.1016/j.neubiorev.2020.03.033)

Response: Individual mice do show a large amount of variance in how much they interact with the device from day to day. The standard deviation of within-mouse usage of the task (across all days of Experiment 2) ranges from 30 - 55 minutes and is close to 50% of mean task usage for any given mouse. However, it is also true that certain mice spend less time than others interacting with the task on average (mean time per day for each mouse across Experiment 2 ranged from 55 - 114 minutes). We observed that the mean time that mice spent in the HASRA was positively correlated with their mean success across days on the task. This could suggest that mice that spend more time practicing the task tend to get better at the task, or perhaps that mice that are more successful at the task tend to engage with it more due to the reinforcing properties of the millet seed reward. We thank the reviewer for their suggestion to investigate the influence of social hierarchies in task usage and will consider this for future experiments. At this time, we believe that it would be more appropriate to explore this question in a dedicated study where we could also record and track the activities and behaviour of each animal towards one another within the homecage, in addition to their interaction with the HASRA. Unfortunately, we do not have this level of data available in the present study.

Minor:

- The reaching task that is being automated is known as the “Single Seed Reaching Task” (Xu et al., 2009, Nature, doi:10.1038/nature08389). It is fine with this reviewer if the authors instead refer to it as a single pellet task, but they should make clear in the Intro that their meaning of this includes the single seed variation.

Response: We thank the reviewer for this comment, but we decided to keep the original title of “single pellet reaching task” as our paradigm was inspired by the original “single pellet task” developed by Dr. Ian Whishaw. However, to clarify that in the present study we do indeed deliver seeds as the item, we have also provided the suggested reference to the seed variant of the task in the Introduction.

- It is not mentioned until deep in the methods (p. 8) that the automated task permits control of the reaching side (left vs. right forelimb). Because the ability to control that is crucial for many applications, the authors should consider making a much more prominent point of this capacity, e.g., in the Abstract. It also doesn’t hurt to highlight it given that the strategy for controlling it is quite clever.

Response: We have emphasized in the abstract and significance statement how the HASRA permits control of item delivery to either reaching side for individual mice. We agree with the reviewer that this is a unique and highly useful element of the design that should be brought to the reader’s attention as quickly as possible within the manuscript.

- The meaning of active arm time needs clarification. Does this mean the time during which the arm was presenting a pellet? It would also be appropriately to more prominently consider the extent to which this reflects task engagement.

Response: We have clarified in the caption for Figure 2 that “active arm time” corresponds to time in which the arm was presenting pellets, and our rationale for using this metric. The reason for using this measure was to avoid counting time in which the animal rapidly entered and exited the tube without waiting for the pellet hopper and arm to move into position and begin presenting pellets. Once the first pellet presentation within a given trial had begun, all time was counted until the mouse exited the apparatus. We consider that only trials in which an animal stayed focused on the task long enough to actually be delivered at least one pellet are reflective of “task engagement”.

- What age were the mice?

Response: Mice were between 44 and 80 days old at time of introduction to the HASRA. This detail has been added to the “Validation experiment design” section.

- The three “Training stages” are presented followed by explanation of how they are being controlled (“Daily qualitative scoring”). To avoid leaving the reader to wonder about the latter while reading the former, the authors might either flip the order of these sections or mention early in the “Training stages” section that the transitions between them were controlled by an experimenter based on video monitoring.

Response: We agree that the narrative sequence of the Methods is improved by switching the position of the “daily qualitative scoring” and “training stages” sections and have amended the manuscript accordingly.

- What was the rationale for the specific locus of the photothrombotic infarcts? Where within motor cortex the infarcts were centered should be mentioned (since “motor cortex” is a big place).

Response: The locus of photothrombotic infarcts was targeted to damage the forelimb motor area of the mouse as previously mapped in Tennant et al., 2011. The rationale for this was to target the specific motor region associated with reaching and grasping, which is the primary motor domain required to successfully retrieve seeds from the HASRA. We have provided additional data and representative images in Figure 5C,D in order to provide much greater information on lesion location to the reader. These figure panels demonstrate the lesions we produced indeed overlap with the forelimb motor representation defined in Tennant et al., 2011. Details regarding this rationale have also been added to the “Photothrombotic stroke induction” section of the Methods.

- In the Introduction: “individual differences between experimenters has a significant effect on the quality of training and overall reaching success rate that is achieved”. This phrasing needs to be adjusted to suggest the effect as a possibility rather than a given, since other studies have failed to detect significant experimenter effects.

Response: We have rephrased the indicated sentence in the introduction to clarify that experimenter-specific differences in training are a possibility of manual training, rather than a guarantee.

- In the following sentence, “...when this criterion is met significant differences can be observed across groups of subjects.”. If the authors are referring to animal batch effects here, this is of unclear relevance to the focus since removal of experimenter contributed variance doesn’t prevent batch effects.

Response: The reviewer is correct that batch effects in animals can still occur regardless of removing experimenter-induced variance. We have removed the indicated part of the Introduction, as we agree with the reviewer that this idea is not relevant to the present experiment.

- The authors might tone down statements regarding experimenter time costs of traditional reaching tasks (∼10 min/day/mouse). A bigger advantage of the automated task could be that it increases the amount of time that mice are engaged in the task per day.

Response: We have rephrased several sentences in the Introduction to emphasize that one-on-one training limits the amount of time that can be devoted to any particular animal (in the interest of training a suitable number of total animals for a given study). We agree with the reviewer that this is an important distinction from reducing the experimenter time cost of traditional reaching tasks. This benefit of the HASRA is also further discussed in the Discussion section.
