# Supplementary material for: The Home-Cage Automated Skilled Reaching Apparatus (HASRA): Individualized Training of Group-Housed Mice in a Single Pellet Reaching Task
Source: eNeuro. 2020 Oct 12;7(5):ENEURO.0242-20.2020. doi: 10.1523/ENEURO.0242-20.2020 (PMC7581188; doi:10.1523/ENEURO.0242-20.2020)
Supplement: Extended Data 1 — Archive of assembly instructions, Python/Arduino code, 3D printing STL files, and wiring diagrams. See “homecage assembly manual.pdf” and “README.md” contained in archive for more details. Download Extended Data 1, ZIP file. [file enu-eN-MNT-0242-20-s03.zip › Extended Data 1/Homecage assembly manual.pdf]

Homecage Assembly Manual

Prerequisites:

1. 3D Printed Parts

   Arm

   Hopper

   Light Holder

   Beam Breaker Mount

   Motor coupler

   Moving stage top

   Standard

   Moving stage bottom

   Tube holder(Camera holder / living room)

   All the parts can be downloaded by this link:

https://drive.google.com/drive/folders/171QfuPhz_QIpAmTQq7dSlA7sy8fYZyb8?usp=sharing

2. Other Parts

   Arduino Nano x 1

   Stepper Motor Driver x 2

   Buck Converter x 2

   Electrical Switch x 1

Female Power supply connection x 1

Capacitor (Around 33 uf) x 1

Stepper Motorx 2

Servo Motor x 1

Connect Limit Switch x 2

3W IR Light x 2

RFID Reader x 1

Moving Stage x 2

Beam Breaker x 1

Mirror x 1

Square Hollow Tube x 1

Camera x 1

12V Power Supply x 1

Arduino Cablex 2

5 x 7 cm PCB Board x 1

Assemble the electronic components:

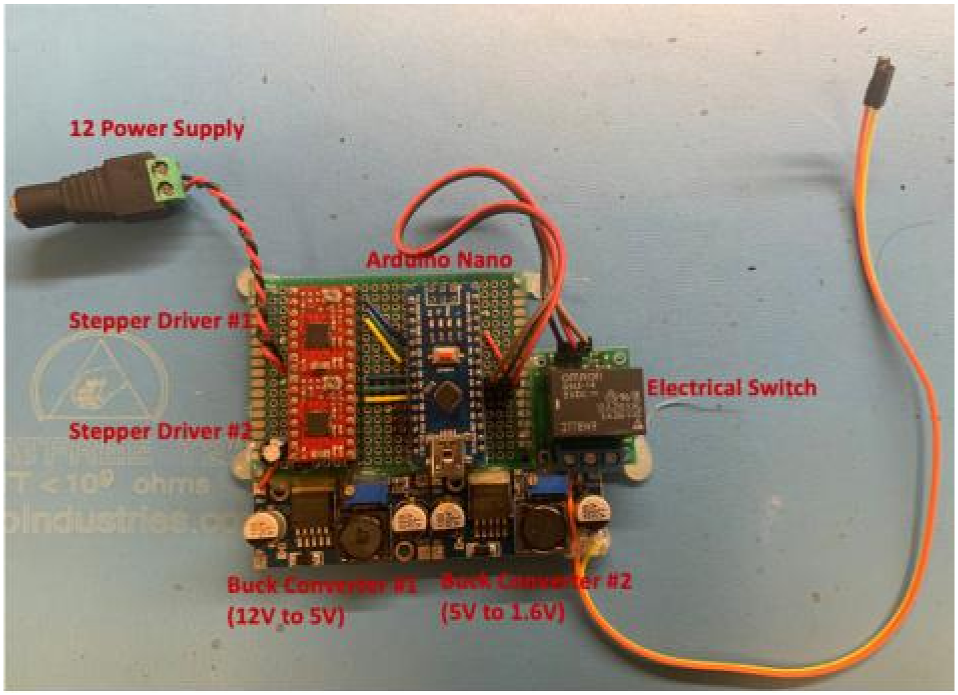

1. Soldering Arduino and stepper motor drivers as the figure above shows, leave 3 lines space at left and right of the circuit board. (do NOT breath in the solder)

2. Adjust the screw on the buck converter to let the output voltage to be 5V (for servo motor).

3. Adjust the second buck converter to let the output voltage to be 1.6V to 1.8V (for IR light).

4. Soldering male headers as shows in red boxes above near D8, D9, D10, A1, A2, A3.

5. Connect the stepper driver pins and Arduino together as the red arrow shows above. (D2, D3, D4 for bottom stepper motor, and D5, D6, D7 for upper stepper motor)

6. As the graph above shows, connect all the left column pins in the left red box to the GND pin on the Arduino.

7. Connect all the left column pins in the right red box to the GND pin on the Arduino. Connect all the middle column pins in the right red box to the GND pin on the Arduino. Connect all the right column pins in the right red box to the 5V pin on the Arduino.

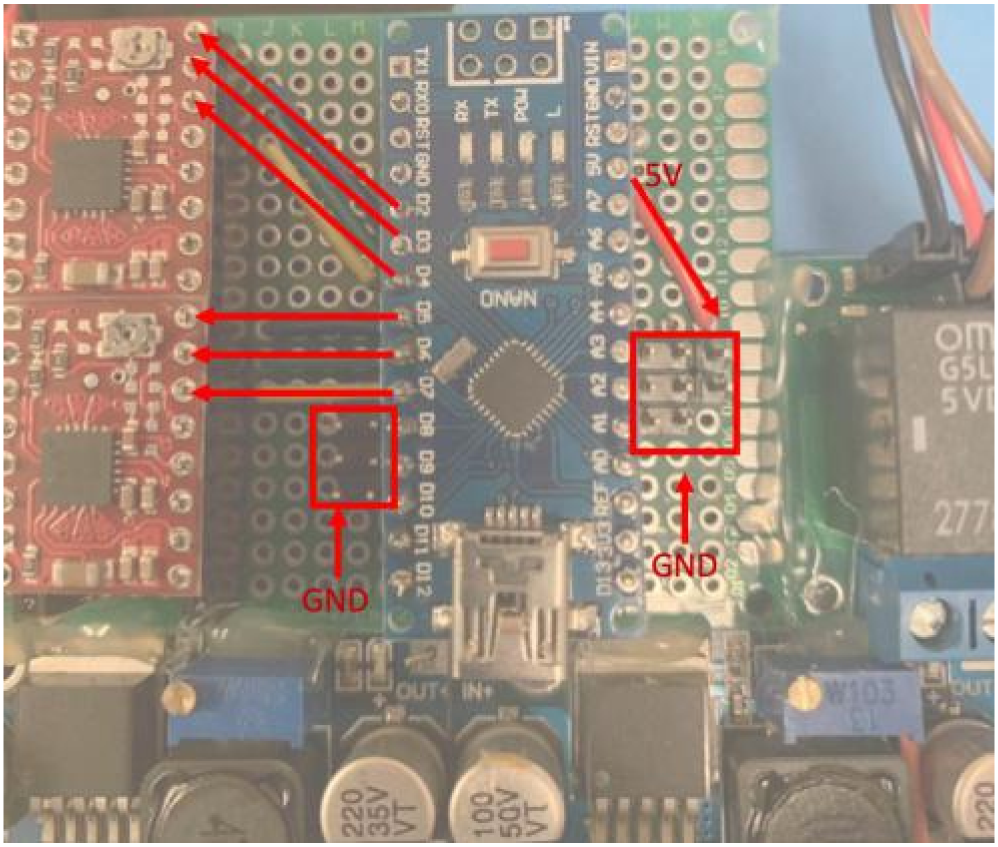

8. Connect the input power to stepper driver as the figure above shows, make these two stepper drivers and the buck convertor #1 to be parallel.

9. Soldering a 33uF capacitor parallel to the power supply.

10. Soldering 4 pins for each stepper driver as the figure above shows in red boxes and connect them to stepper drivers.

11. Connect the positive output of buck converter #1 to a pin near the Arduino pin D8 as the middle red arrow at the figure above shows and connect it to the input of the buck converter #2.

12. Connect the negative output of buck converter #1 to positive input of the buck converter #2.

13. Connect the positive output of buck converter #2 to the right pin on the electrical switch and put a jump wire from the left pin on the electrical switch.

14. Connect a jump wire at the negative output of buck converter #2. (Used to power the IR Light)

15. Connect stepper driver GND to Arduino GND. Check to make sure all grounds are connected.

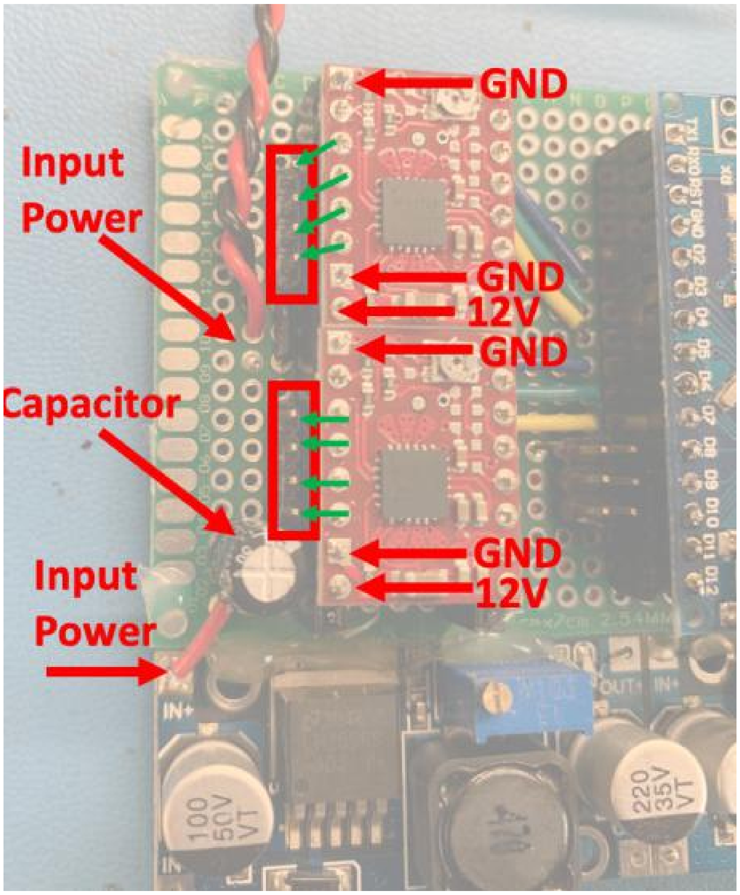

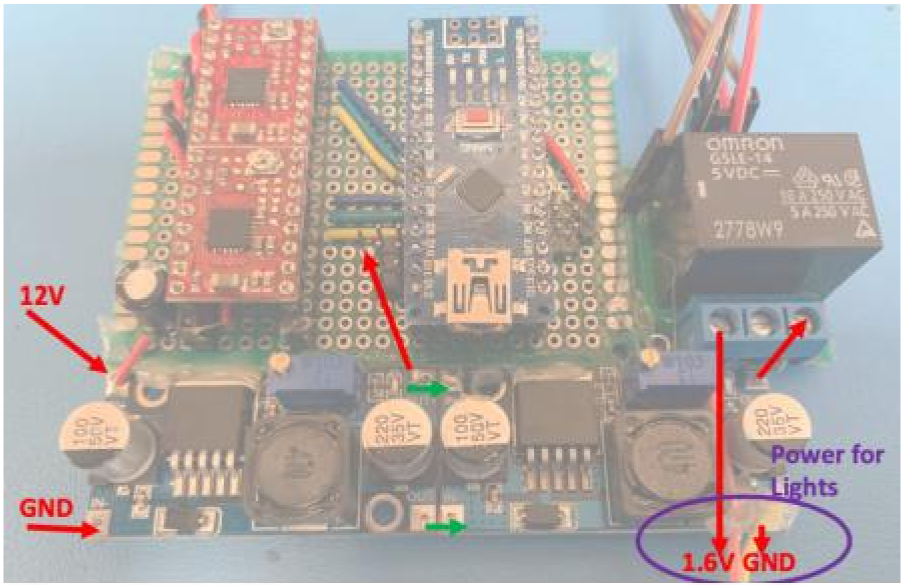

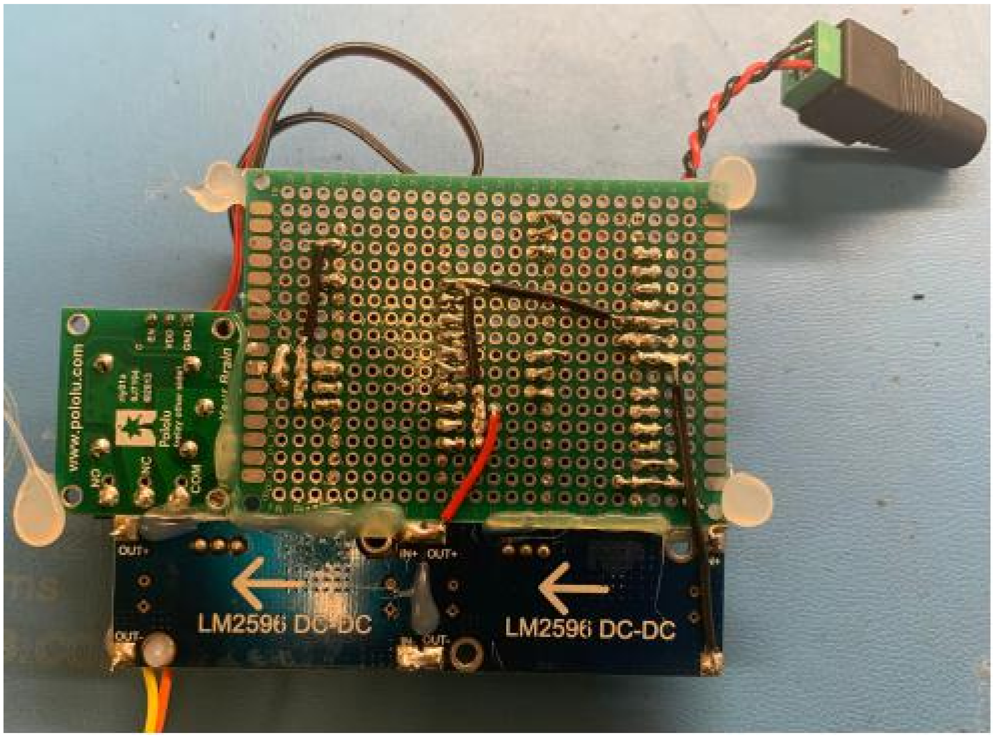

Assemble the mechanical components:

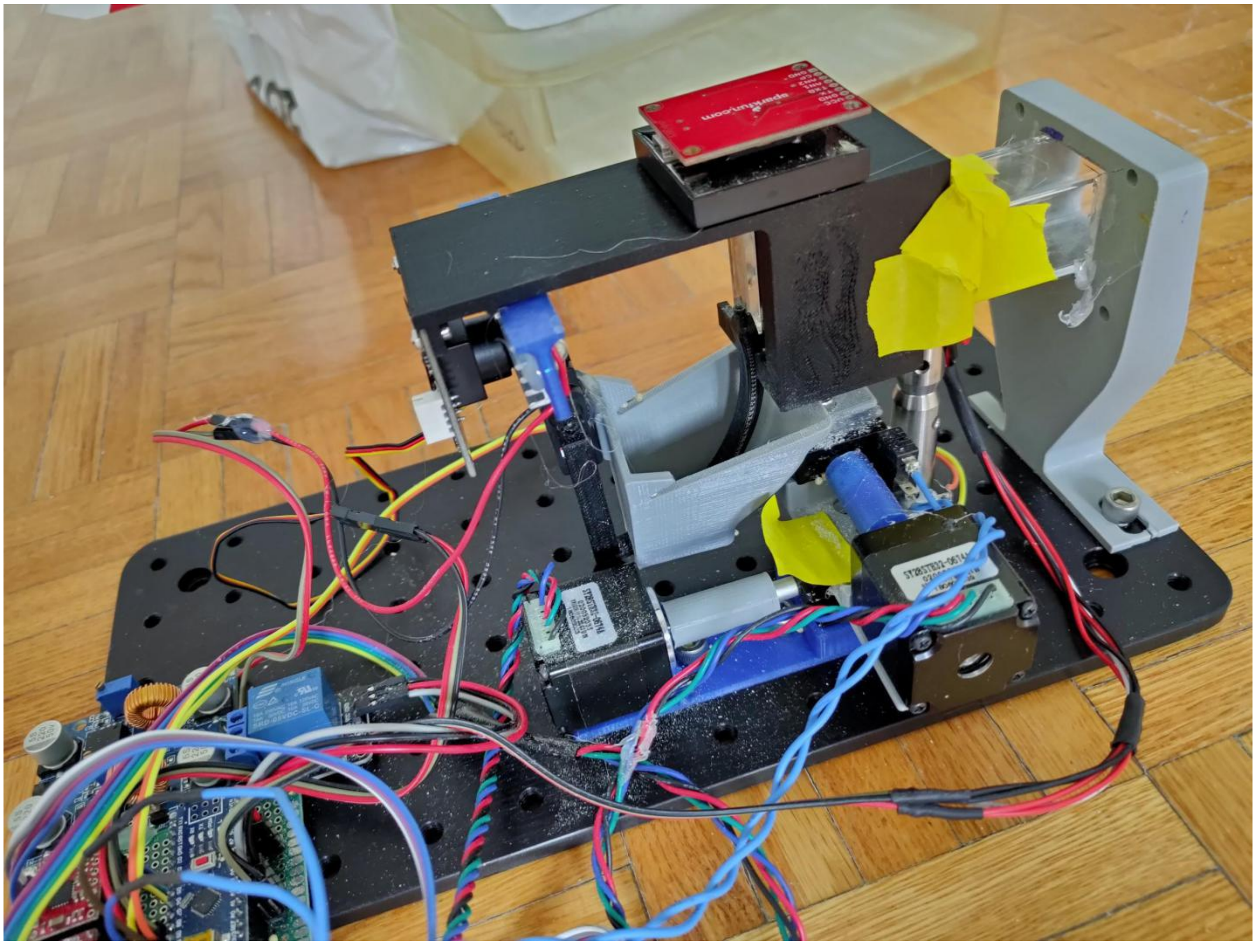

1. Camera Holder Module

1.1 Tube and support

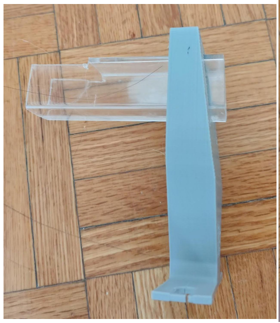

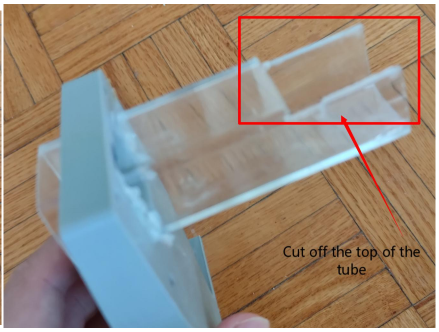

Insert the tube into the 3d printed support. The top of the front part of the tube needs to be cut off.

1.2 Camera holder(Living room)

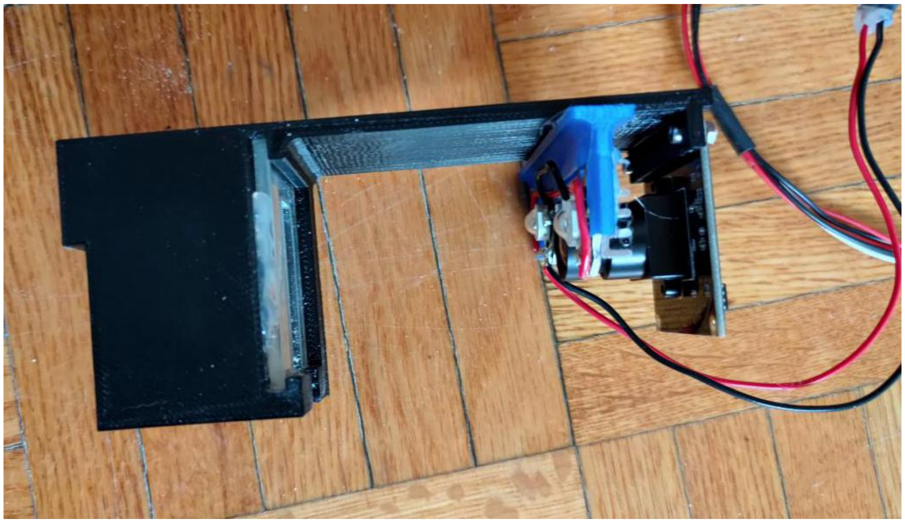

1.2.1 Glue the light holder to the roof as shown below:

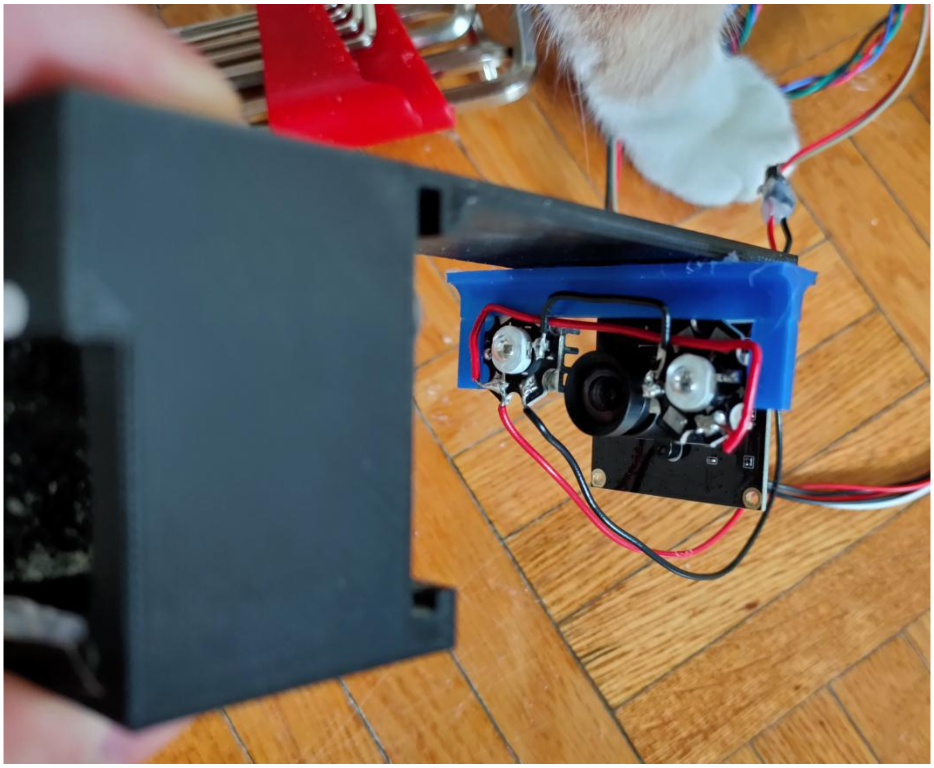

1.2.2 Attach the camera to the camera holder(living room) by placing the screws and nuts

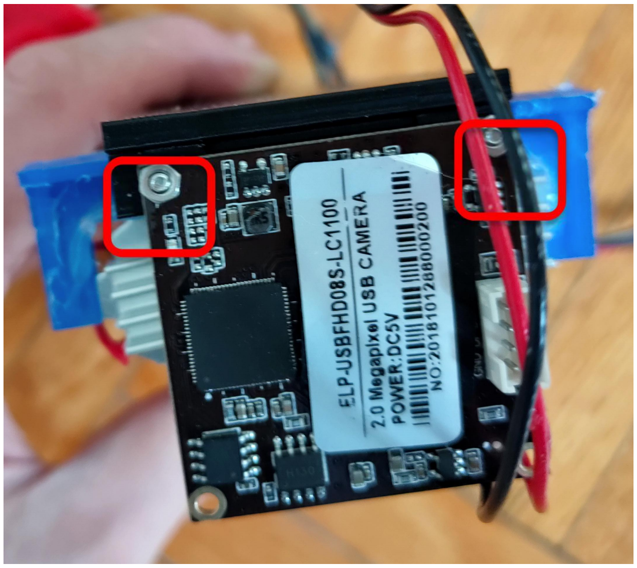

1.2.3 Insert the replaceable Acrylic front piece

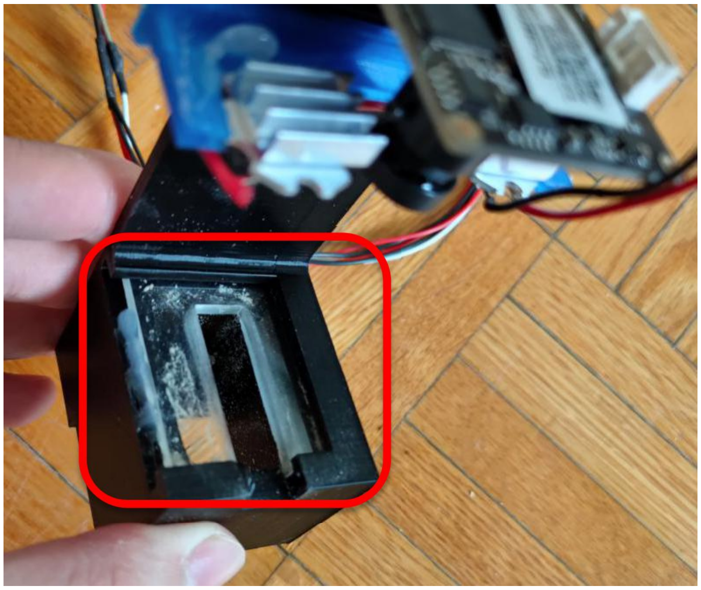

1.3 Attach the RFID Reader

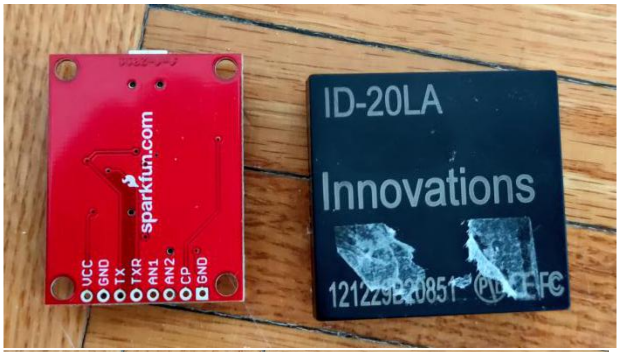

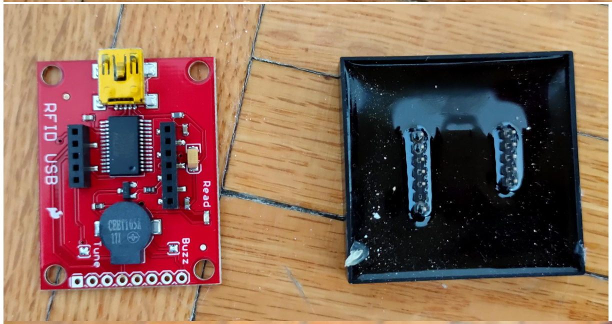

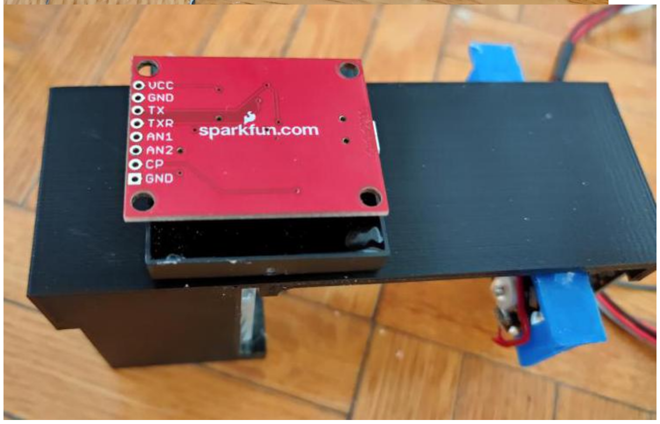

1.4 Attach the beam breaker

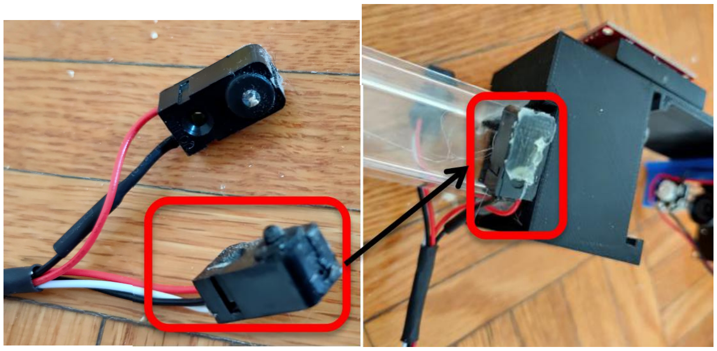

Tape and anything could block the light are strongly recommended to apply here. Sometimes the beam breaker cannot detect the mouse, because the receiver is continuously triggered by external light source.

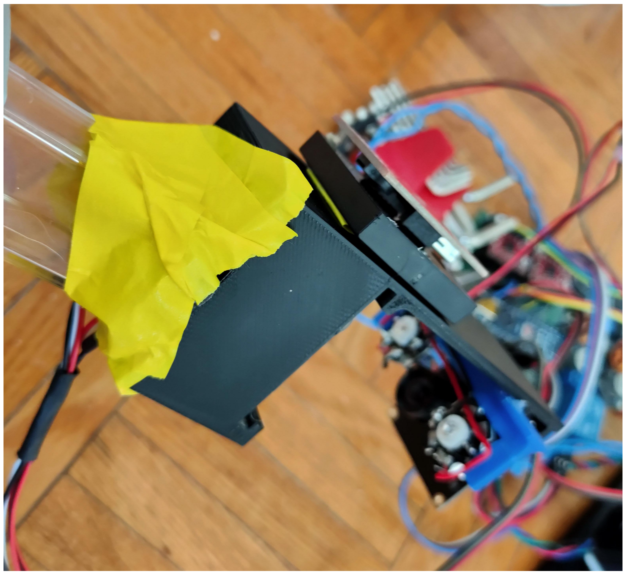

1.4 Fit the tube into the camera holder(living room)

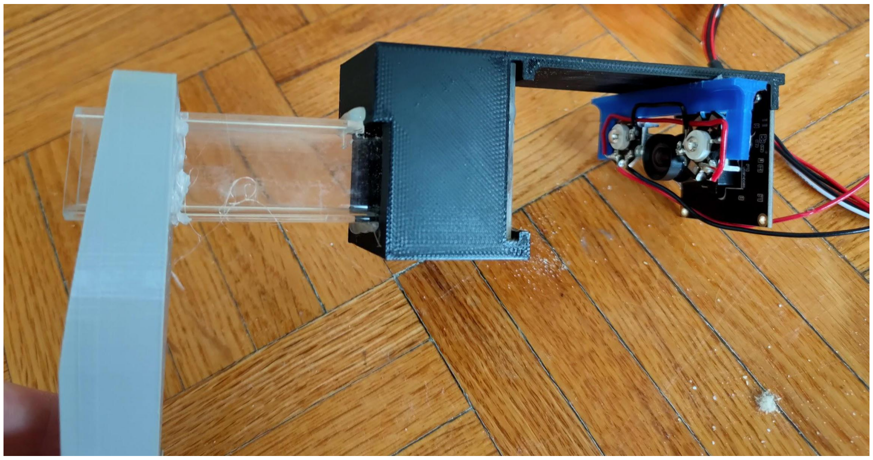

2. Motor Module

2.1 upper stage(controlling left and right)

Glue the switch like this:

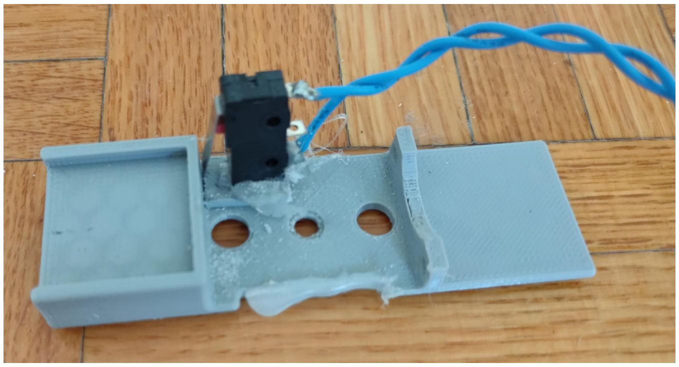

All the parts we need for this stage.

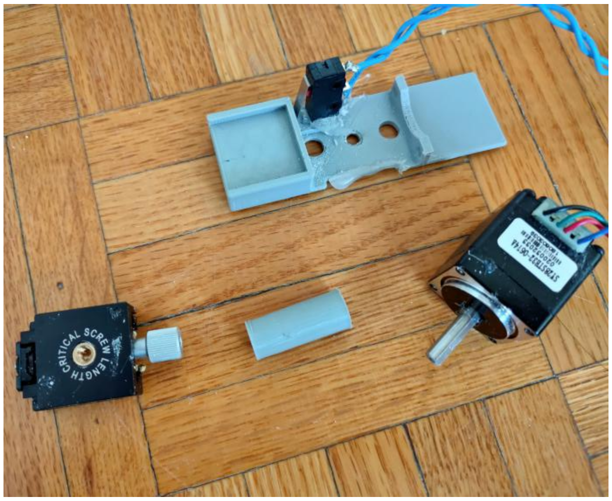

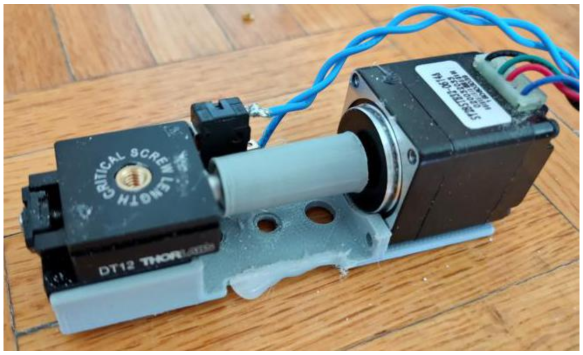

Tips: make sure the switch can be triggered correctly as shown below.

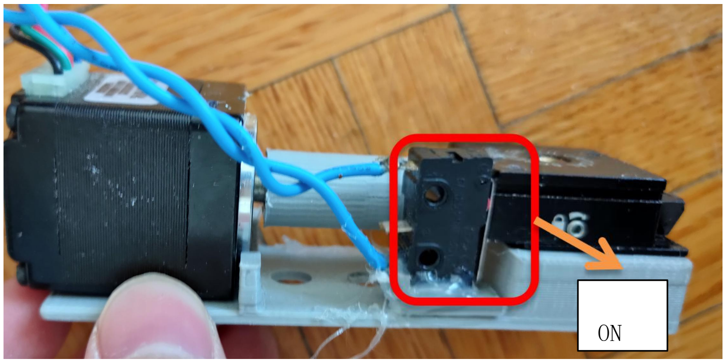

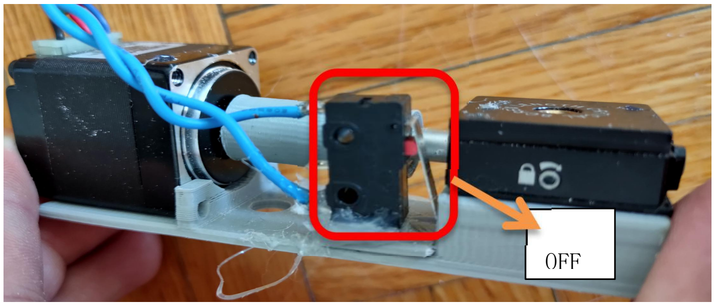

Use screws to attach the motor to the 3d printed stage.

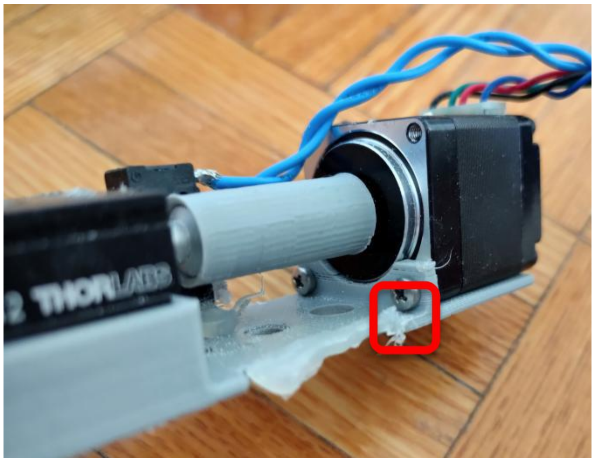

2.2 Lower stage(Front and back)

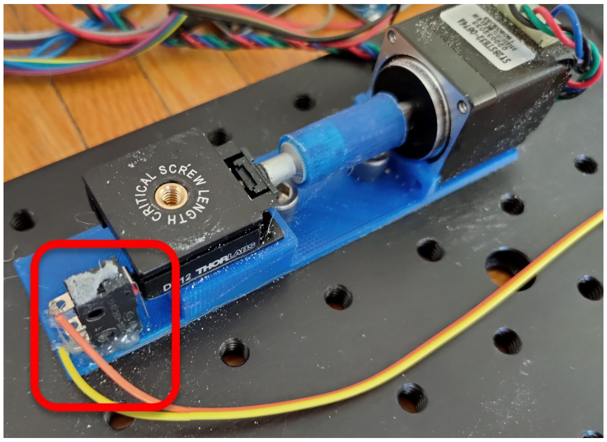

Only the position of the switch is different. Make sure it is screwed on the metal board.

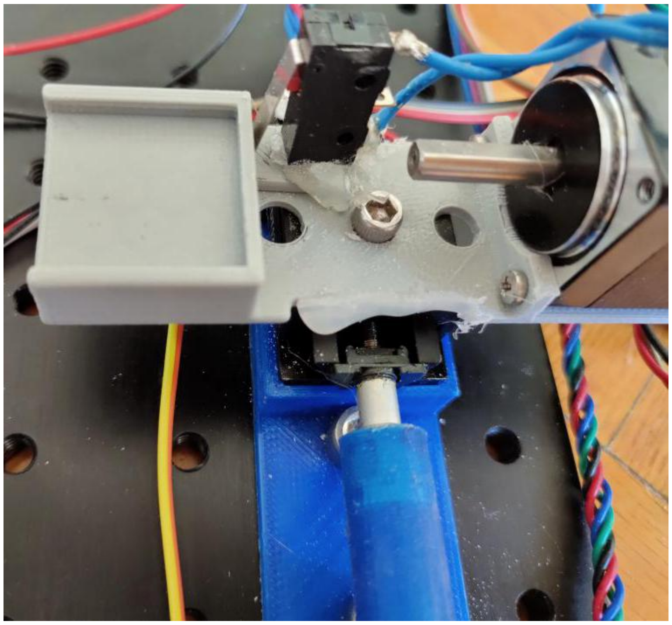

Place the upper stage on the top of lower stage.

2.3 Attach the servo motor and the arm to the hopper

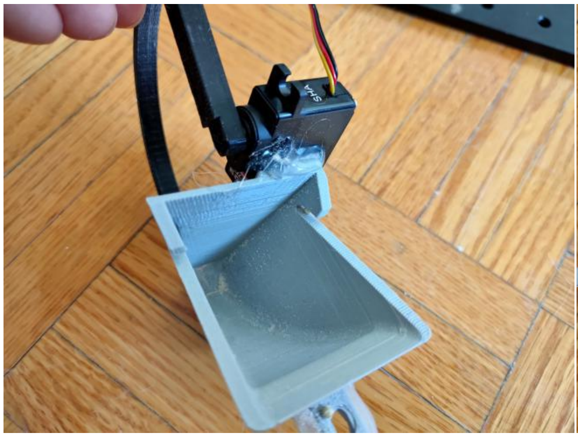

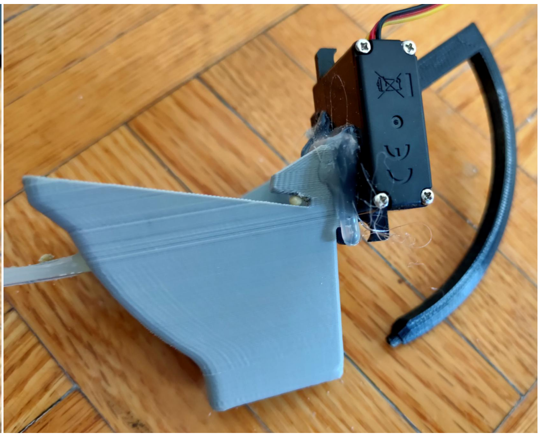

Note that you need to find a proper position before you glue the servo motor. Test it by moving the arm manually, make sure it is smooth.

Then attach the whole part to the upper stage.

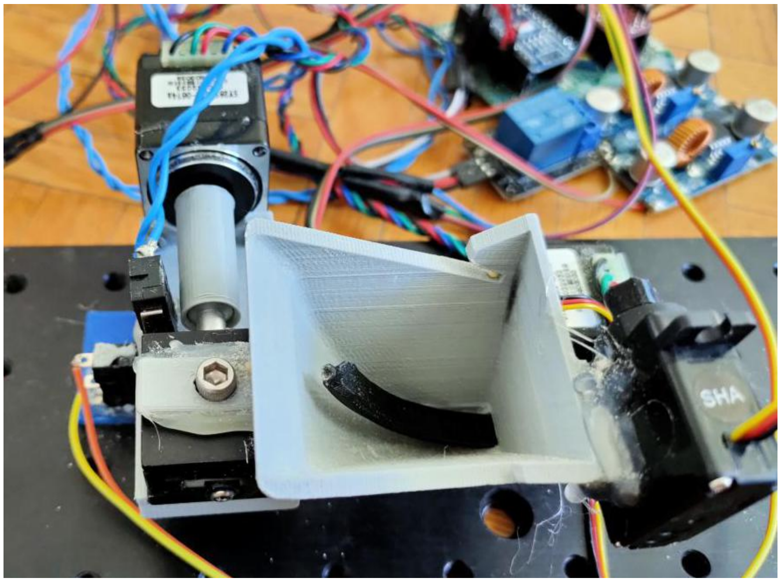

2.4 Add an additional support

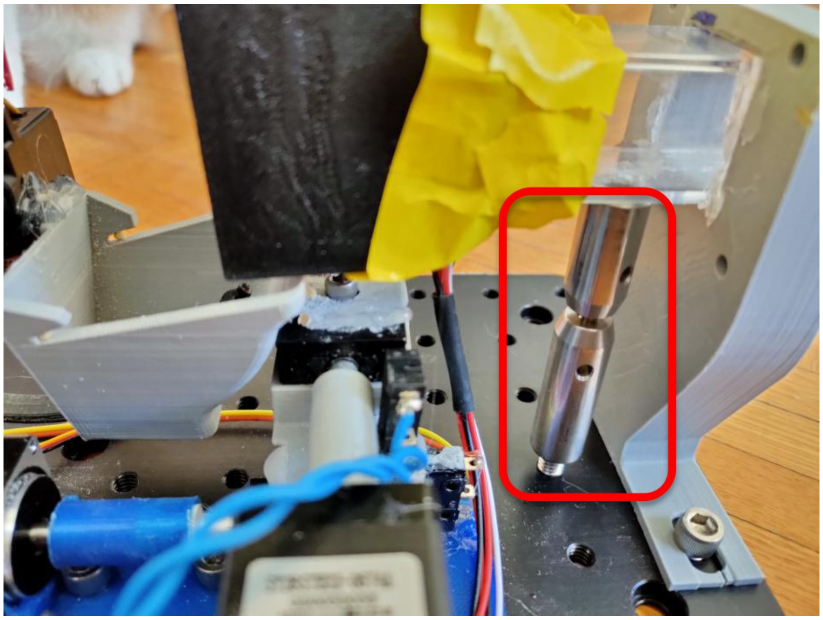

By doing this, you can make sure the whole system is working with sufficiently stability.

Connect Components on the PCB Board:

Simple and easy.

- Connect all the component to it's right pin.
- Distinguish signal pins, GND pins, 5v pins
- The beam breaker and the electrical switch use the 5v power from the Arduino, and the servo motor use the 5v power from buck converter #1.

Check the figure below for other details.

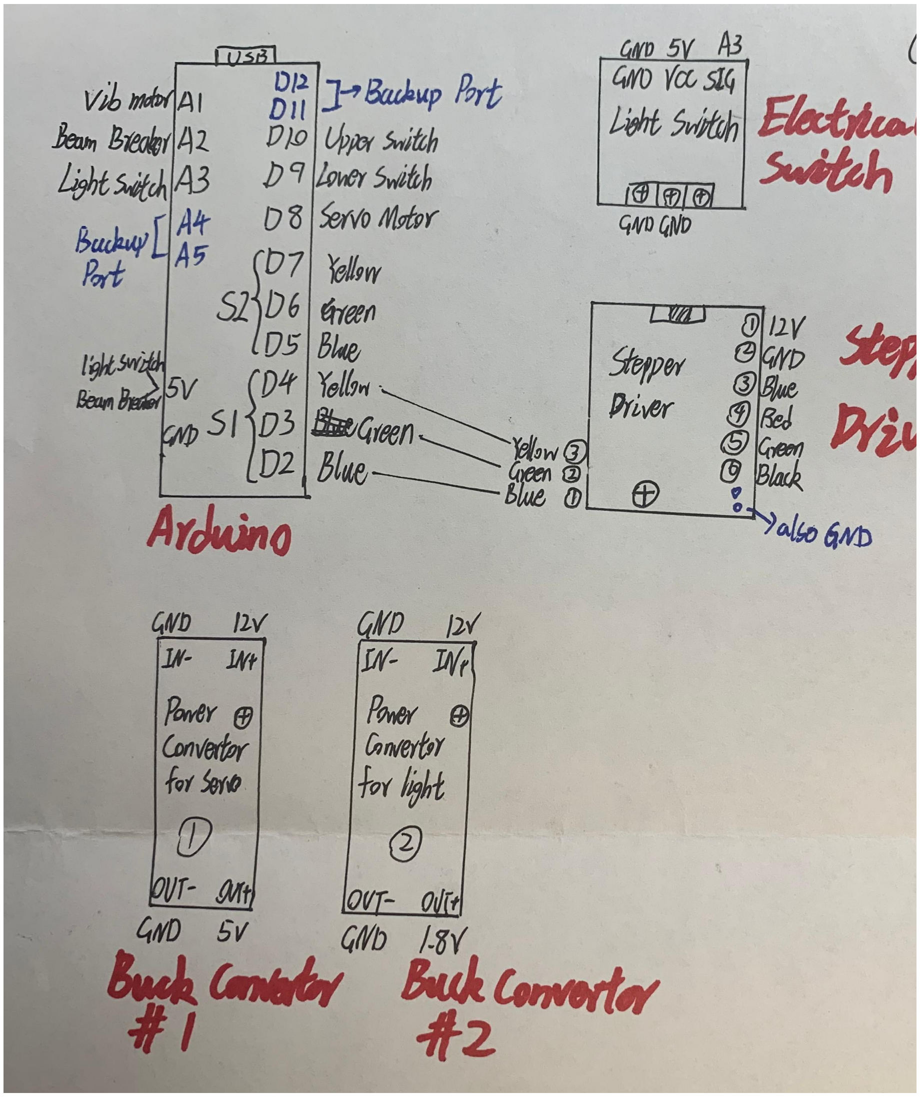

USB
D12
D11
Backup Port
Vib motor A1
Beam Breaker A2
Light Switch A3
Backup Port
A4
A5
D10 Upper Switch
D9 Lower Switch
D8 Servo Motor
D7 Yellow
D6 Green
D5 Blue
S2
light switch
Beam Breaker
5V
GND
S1
D4 Yellow
D3 Green
D2 Blue
Arduino
GND 5V A3
GND VCC SIG
Light Switch
GND GND
Switch
Stepper Driver
12V
GND
Blue
Red
Green
Black
Yellow
Green
Blue
also GND
GND 12V
IN- IN+
Power Converter for Servo
OUT- OUT+
GND 5V
Buck Convertor #1
GND 12V
IN- IN+
Power Convertor for light
OUT- OUT+
GND 1.8V
Buck Convertor #2
