# Supplementary figures and images for: The Home-Cage Automated Skilled Reaching Apparatus (HASRA): Individualized Training of Group-Housed Mice in a Single Pellet Reaching Task
Source: eNeuro. 2020 Oct 12;7(5):ENEURO.0242-20.2020. doi: 10.1523/ENEURO.0242-20.2020 (PMC7581188; doi:10.1523/ENEURO.0242-20.2020)

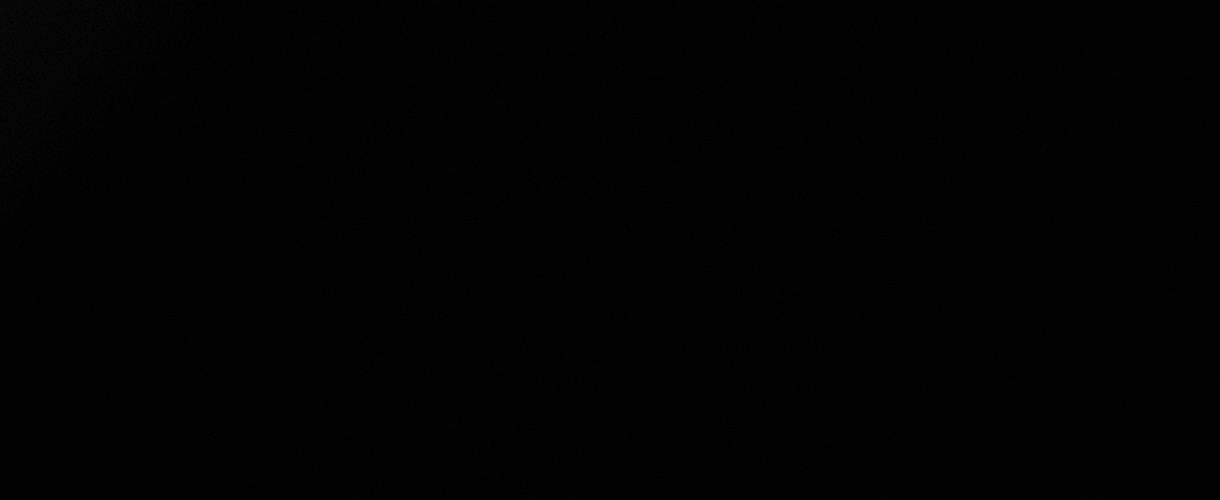

Supplement: Extended Data 1 — Archive of assembly instructions, Python/Arduino code, 3D printing STL files, and wiring diagrams. See “homecage assembly manual.pdf” and “README.md” contained in archive for more details. Download Extended Data 1, ZIP file. [file enu-eN-MNT-0242-20-s03.zip › Extended Data 1/temp/pelletClassifierFatMouse.jpg]

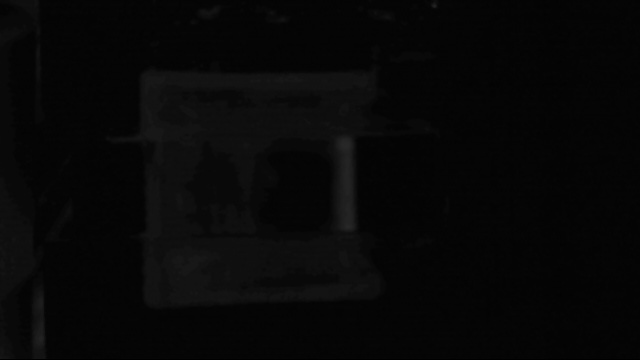

Supplement: Extended Data 1 — Archive of assembly instructions, Python/Arduino code, 3D printing STL files, and wiring diagrams. See “homecage assembly manual.pdf” and “README.md” contained in archive for more details. Download Extended Data 1, ZIP file. [file enu-eN-MNT-0242-20-s03.zip › Extended Data 1/src/client/detection_frame.jpg]
